# Heterologous Antigen Regimen and Bicistronic DNA Vaccination Targeting HPV16 E5 and E7 Oncoproteins: Immunoinformatics and Preclinical Antitumor Efficacy

**DOI:** 10.3390/molecules31193442

**Published:** 2026-09-27

**Authors:** Ingrid Andrêssa de Moura, Karina Mayumi Tani Bezerra de Melo, Lígia Rosa Sales Leal, Maria da Conceição Viana Invenção, Larissa Silva de Macêdo, Samara Sousa de Pinho, Lucas Matheus Barreto Santana, Julia Dias da Silva, Tiago Henrique dos Santos Souza, Julliano Matheus de Lima Maux, Jacinto da Costa Silva Neto, Anna Jéssica Duarte Silva, Antonio Carlos de Freitas

**Affiliations:** 1 Laboratory of Molecular Studies and Experimental Therapy—LEMTE, Department of Genetics, Federal University of Pernambuco, Recife 50670-901, Brazil; ingrid.andressa@ufpe.br (I.A.d.M.); karina.mayumi@ufpe.br (K.M.T.B.d.M.); ligia.leal@ufpe.br (L.R.S.L.); maria.conceicao@ufpe.br (M.d.C.V.I.); larissa.smacedo@ufpe.br (L.S.d.M.); samara.pinho@ufpe.br (S.S.d.P.); julia.diass@ufrpe.br (J.D.d.S.); anna.jessica@ufpe.br (A.J.D.S.); 2 Department of Virology and Experimental Therapy, Fundação Oswaldo Cruz/Fiocruz, Recife 50740-465, Brazil; lucas96barreto@gmail.com; 3 Department of Biophysics and Radiobiology, Federal University of Pernambuco, Recife 50670-901, Brazil; tiago.henriques@ufpe.br; 4 Laboratory of Cytological and Molecular Research—LPCM, Department of Genetics, Federal University of Pernambuco, Recife 50670-901, Brazil; julliano.maux@ufpe.br (J.M.d.L.M.); jacinto.costa@ufpe.br (J.d.C.S.N.)

**Keywords:** therapeutic DNA vaccine, HPV16, E5 oncoprotein, E7 oncoprotein, nitric oxide, histopathology, prime–boost immunization, murine model

## Abstract

Persistent infection with high-risk human papillomavirus, particularly HPV16, is a major cause of cervical cancer and orogenital malignancies. Therapeutic DNA vaccines targeting viral oncoproteins represent a promising strategy to induce antitumor immune responses. However, the impact of antigen combinations in bicistronic vectors and the immunization sequence on therapeutic efficacy remains poorly understood. Thus, this study aimed to characterize in silico previously described E5CP and E7CP vaccines, develop a new bicistronic construct, and evaluate its antitumor efficacy under different immunization regimens in vivo. The constructs were initially characterized regarding predicted physicochemical, immunological, and structural properties, followed by molecular confirmation of plasmid construction and qualitative assessment of transcript detection after transient transfection. In vivo, C57BL/6 mice bearing C3 tumors were immunized with five different regimens combining the monocistronic or bicistronic constructs. At the endpoint, tumors and spleens were collected for histopathological and immunological analyses. The results showed that regimens using the bicistronic construct or including E5 in the first dose were associated with lower mean tumor volumes or delayed tumor growth compared to the control groups. Histopathological and immunophenotypic changes were variable among regimens, and an exploratory analysis suggested an association between lower nitric oxide levels and smaller tumor volumes. Therefore, the order of antigen administration and the use of bicistronic constructs warrant further investigation as strategies that could be optimized to improve the therapeutic efficacy of HPV DNA vaccines.

## 1. Introduction

Cancer is a multifactorial disease that can arise from genetic and environmental factors and whose incidence is reaching increasingly pronounced levels of occurrence and mortality [1,2]. Among environmental factors, infections are associated with 15% to 20% of cancer cases, the majority of which are linked to oncogenic viruses [3,4]. This rate is even higher in developing countries, where adherence to preventive, sanitary, and screening measures is low [5,6].

Viruses are capable of regulating growth pathways, apoptosis, and cell proliferation and of directly impacting the genome of human cells through gene dysregulation, the occurrence of somatic mutations, and the integration of their genetic material into the host cell’s DNA [3,6]. Among them, human papillomavirus (HPV) is responsible for 5% of cancers [7,8], with a focus on cervical cancer, anogenital cancers, and oropharyngeal cancers [9,10,11]. Despite there being more than 200 cataloged genotypes, high-risk types 16 and 18 are the most prevalent, accounting for approximately 70% of all cervical cancer cases [12,13,14].

HPV-induced carcinogenesis is caused by the ongoing expression of the viral oncogenes E6 and E7 from high-risk types, which is often linked to the degradation of the tumor suppressors p53 and pRB [15]. In high-risk HPVs, E5 also participates in evading the immune response, reduces cellular dependence on external growth factors, and promotes cellular proliferation, migration, and invasion [16]. Its contribution to carcinogenesis is more relevant in the early stages of infection, while the maintenance of the malignant phenotype mainly depends on the continuous expression of E6 and E7 [17,18]. Despite preventive vaccines and screening strategies having significantly contributed to the reduction in HPV-associated malignancies, their acceptance is still limited, and they do not treat already developed lesions and tumors [19,20]. For these individuals, especially in more advanced stages, conventional treatments are based on invasive methods with severe adverse effects and recurrences [21,22,23].

In this context, DNA therapeutic vaccines that have the ability to induce CD8^+^ and CD4^+^ T cell responses emerge as a promising approach for the treatment of HPV-associated cancers [24,25]. However, this platform is associated with scientific challenges that limit its translation and approval by regulatory agencies. Among them are the need to optimize delivery techniques, increase immunogenicity, select new antigens, and establish an immunization regimen [26,27].

To increase immunogenicity and select new antigens, previous studies have shown that DNA vaccines encoding HPV16 antigens fused to the capsid protein of potato virus X (PVX) have therapeutic potential [28,29,30]. In particular, Paolini et al. (2017) [30] reported that homologous regimens with E5 or E7 fused to the CP capsid protein, given in four doses of 100 μg, significantly controlled tumor growth in mice challenged with C3-Luc cells. The construct pVAX E5CP, in particular, increased immunogenicity and induced a strong CD4^+^ T cell response, leading to 80% regression in a murine model of HPV16+ tumors, while pVAX E7CP induced complete regression in about 40% [30]. However, despite these results, it is still unknown whether strategies with regimens that combine different HPV antigens on the same platform could increase therapeutic efficacy and broaden the repertoire of T cell responses. Moreover, previous studies have only evaluated monocistronic vaccines, leaving unexplored whether the simultaneous expression of E5 and E7 from a single DNA construct could enhance antitumor immunity. In this context, bicistronic DNA vaccines represent an attractive alternative, coordinating the expression of multiple antigens within a single plasmid structure, which can enhance immune recognition and simplify vaccine administration [27].

Given the above, the present study aimed to (i) perform the in silico characterization of the previously described monocistronic constructs pVAX-E5CP and pVAX-E7CP; (ii) develop a new bicistronic construct pVAX-HisE5CP–IRES–HaE7CP; and (iii) evaluate the therapeutic potential of these vaccines in different immunization regimens in a murine model of an HPV-16-positive tumor.

## 2. Results

### 2.1. Prediction of Physicochemical Properties, Toxicity, and Allergenicity of Chimeric Proteins

The physicochemical, toxicological, and allergenic properties of the chimeric proteins were predicted using the servers described in Section 4. Table 1 summarizes the key points of each prediction.

The ExPASy ProtParam tool (https://web.expasy.org/protparam/) [31] results showed that HisE5CP and HaE7CP are potentially stable proteins but present distinct physicochemical profiles. The chimeric protein HisE5CP has a higher aliphatic index, is more hydrophobic, and has a theoretical pI of 8.09, consistent with a predominantly basic character. Conversely, HaE7CP is more hydrophilic and predominantly acidic, with a higher number of cysteine residues. Regarding the safety profile, although four and thirteen potentially toxic peptides were identified within the HisE5CP and HaE7CP sequences, respectively, the full-length proteins were classified as non-toxic by the ToxinPred 2.0 tool and probably non-allergenic by AllerTOP 2.0 (Supplementary Table S2).

### 2.2. Analysis of RNA Secondary Structure

The secondary structures of the mRNAs were predicted using the RNAfold tool. Table 2 summarizes the thermodynamic parameters obtained for each construct, whereas Figure 1 presents the secondary structures predicted based on the full-length mRNA sequences.

Secondary structure prediction showed that all three transcripts had negative MFE values, ranging from –289.40 kcal/mol (HisE5CP) to –886.20 kcal/mol (bicistronic), with high conformational diversity (170.30–575.40) and 0% MFE frequency for all constructs (Table 2, Figure 1). These parameters indicate that the mRNAs adopt a broad ensemble of structures rather than a single rigid conformation. In addition, the difference between the MFE and the ensemble free energy was more pronounced for the bicistronic construct (Δ = 37.50 kcal/mol) than for the monocistronic constructs (Δ ≈ 15–16 kcal/mol). This implies that the minimum-energy structure is particularly unrepresentative for the bicistronic mRNA, and the centroid structure must be considered the most thermodynamically relevant state (Figure 1C). These predictions suggest that the bicistronic mRNA could form several alternative secondary structures with considerable conformational heterogeneity. However, they do not constitute direct evidence for IRES function or translational efficiency.

### 2.3. Prediction of Three-Dimensional Structures, Overall Quality Assessment, and Refinement

To predict the 3D structures of the E5 and E7 constructs fused to CP, we used AlphaFold 3 [32]. The results demonstrated high prediction accuracy for the CP and E7 elements (plDDT > 90), whereas E5 showed intermediate confidence. Tags and the connection regions between the oncoproteins and the fused adjuvant protein showed plDDT < 50 (Figure 2). The obtained pTM values (0.57 for HaE7CP and 0.60 for HisE5CP) reflect a moderate-to-low confidence in the predicted global folds. Consequently, these structural models function as computational hypotheses rather than experimentally validated conformations.

After structural prediction by AlphaFold 3, the constructions were evaluated in ModFold8 to validate the global quality of the models (Table 3) [33]. Based on confirmation of the model’s validity, the structures were subjected to analysis of contact between atoms, conformational stability, and possible rotamers by the MolProbity web service [34]. Therefore, to investigate whether we could further improve model quality, we refined the original AlphaFold3 file using the ReFOLD3 server [35]. However, comparative analysis of the models indicated that the refinement process, despite increasing the global model quality score, introduced problems with stereochemical parameters (Figure S1, Table S3, Supplementary Materials). Therefore, the original AlphaFold3 models were retained as the reference computational models for subsequent structural visualization, while acknowledging the limited confidence associated with their global conformations.

### 2.4. MHC Preservation of Immunologically Relevant Epitopes Following Fusion to CP

To investigate whether fusing HPV16 E5 and E7 to the CP could affect antigen processing and presentation, we performed MHC-I binding and processing predictions for the native antigens and their respective fusion proteins (Tables S4–S7, Supplemental Material). The results show that incorporating the CP molecular adjuvant did not alter the presentation potential of the major cytotoxic T-cell epitopes for classical alleles in C57Bl/6 mice (Table 4). For both HPV16 E5 and E7, the most immunologically relevant epitopes remained conserved after fusion to CP, exhibiting identical percentile ranks and processing scores. Among the strongest predicted binders, the E5 epitopes IIFVYIPLF, LSVSTYTSL, STYTSLIIL, and IVYIIFVYI had percentile ranks below 0.2 for the H2-Kb allele, indicating high predicted affinity. In addition, the experimentally validated epitopes VCLLIRPLL and YIIFVYIPL [36,37] were also preserved after fusion to CP. These epitopes were included for reference despite their percentile ranks being above the in silico selection threshold, as they have been previously validated in the literature and were selected for ex vivo restimulation independently of the in silico criteria. The experimentally validated HPV16 E7 epitope RAHYNIVTF exhibited the strongest predicted binding, particularly for the H2-Db allele (percentile rank = 0.01).

Despite the complete preservation of MHC-I epitopes, MHC-II epitopes restricted to H2-IAb showed distinct results for E5CP and E7CP (Table 5). For E7CP, almost all helper T cell epitopes identified in the native E7 sequence were retained after fusion with CP, exhibiting identical percentiles. Although the N-terminal peptide MHGDTPTLHEYMLDL was not detected in E7CP, an overlapping peptide, AHGDTPTLHEYMLDL, was identified with a similar percentile and preserved the predicted GDTPTLHEY binding core. On the other hand, the fusion of E5 with CP altered the overall repertoire of MHC-II epitopes. In this case, the recognition core was preserved, indicating the partial preservation of helper T cell determinants. Furthermore, relevant helper epitopes identified in the native E5 sequence were replaced by CP-derived peptides in the fusion construct.

### 2.5. In Silico Immunological Profiling of the E5/E7 Vaccine Constructs

The simulated immune profiles, generated within a human HLA context, reflect qualitative predictions over a 25-day simulation of a two-dose vaccination regimen with the constructs HisE5CP, HaE7CP, and bicistronic HisE5CP-IRES-HaE7CP. The HaE7CP construct elicited the most pronounced response, while the bicistronic vaccine exhibited an intermediate response, and HisE5CP showed the lowest response across the different conditions (Figure 3).

In the CD4^+^ helper T cell population, HaE7CP reached a peak of over 7000 active cells per mm^3^, while HisE5CP induced an expansion similar to that of the bicistronic construct, ~6000 cells per mm^3^ (Figure 3A). The population of CD8^+^ cytotoxic T lymphocytes increased in all groups; HisE5CP and the bicistronic vaccine had nearly identical profiles, both reaching ~600 active cells per mm^3^ on day 25, while HaE7CP showed a slightly earlier onset of activation (Figure 3B). The analysis of antigen-presenting cells showed no significant differences in dendritic cell activation across all constructs, either in activation or antigen presentation (Figure 3C). On the other hand, macrophages exhibited a sharp increase in the presenter-2 subpopulation after bicistronic vaccination, with a pronounced peak above 100 cells per mm^3^ on day 5 and a secondary peak of ~50 cells per mm^3^ between days 10 and 15 (Figure 3D). In addition, all vaccines induced the expression of the cytokines IFN-γ, IL-2, and IL-10, as well as danger signals, with HaE7CP showing the highest amplitude and the bicistronic formulation producing an intermediate yet more sustained response (Figure 3E).

Although these vaccines are intended for therapeutic purposes, C-IMMSIM predicted humoral responses. HaE7CP induced the highest levels of antigen-specific immunoglobulins and B-cell activation, while HisE5CP generated the weakest response, and the bicistronic construct showed an intermediate profile. Supplementary Figure S2 presents detailed predictions of antibody isotypes, B-cell populations, and plasma cell dynamics.

### 2.6. Cloning and Transcript Detection of the Vaccine Constructs

We assessed the molecular validation of the constructs and confirmed their construction by restriction analysis and qualitative PCR, respectively (Figure 4). The enzymes *Hind*III, *Not*I, *Bam*HI, *Xba*I, and *Xho*I were used to release the inserts from the monocistronic and bicistronic vectors according to the in silico cloning design and sequence specificity (Figure S3). The digestions shown in Figure 4A,B display fragment patterns consistent with the anticipated molecular sizes, including the E5, E7, CP, and IRES regions.

Additionally, to assess the constructs’ ability to generate transcripts in vitro, transfected HEK-293T cells underwent RNA extraction, cDNA conversion, and conventional PCR (Figure 4C,D). We used specific primers for E5 and E7 (monocistronic vaccines) and for E5 and IRES-E7 (bicistronic vaccine), as detailed in the Supplementary Materials (Table S1). Amplification products of the expected sizes were detected in all three replicates for both target regions (Figure 4C,D). Conversely, no corresponding amplification was detected in cells transfected with the empty pVAX1 vector or in the negative control.

### 2.7. Protein Expression Analysis by Western Blot

For further verification of protein-level expression of the chimeric constructs, Western blotting was performed on cell lysates from HEK-293T cells transfected with the respective constructs, using anti-polyhistidine and anti-HA antibodies (Figure S4). For E5CP, several bands were detected, including higher-molecular-weight bands corresponding to predicted multimeric forms of E5. In the case of E7CP, several bands were detected, including bands near the predicted molecular weight; however, similar bands were also detected in the empty vector control. This resulted in substantial background that did not allow unambiguous assignment of these bands to E7CP. Therefore, the Western blot results do not provide definitive protein-level confirmation of E7CP expression from the bicistronic construct (Figure S4).

### 2.8. In Vivo Antitumor Efficacy of the E5/E7 DNA Vaccination Strategies

The therapeutic efficacy of the different DNA vaccination regimens was evaluated in the C3 HPV16 tumor model, as shown in the experimental design in Figure 5A. Mice were inoculated subcutaneously with 5 × 10^5^ C3 tumor cells on day 0 and immunized intramuscularly with 50 μg of plasmid DNA, followed by electroporation on days 3 and 10. Animals were monitored throughout the experiment and euthanized on day 27 for tumor and spleen collection.

Tumor incidence increased progressively across all experimental groups during the follow-up period (Figure 5B). Most vaccinated groups reached approximately 100% tumor incidence by day 17. However, there were two losses due to anesthesia in the pVAX control group, which reached complete tumor incidence only at the end of the experiment. Therefore, tumor volume analyses included only the three remaining mice from the pVAX group (Supplementary Table S8).

Tumor growth was monitored throughout the experimental period (Figure 5C,D). Several vaccination regimens, particularly those initiated with E5-containing constructs, showed lower mean tumor volumes or delayed tumor growth relative to the control group at different stages of follow-up. In the E7/E5E7 group, a slight reduction in tumor volume was observed between days 24 and 27; however, this difference did not reach statistical significance. No significant body weight loss was observed in any experimental group throughout the study, indicating good tolerability of the DNA vaccination regimens (Supplementary Table S9).

#### 2.8.1. Histopathological Analysis

The histopathological characteristics of the tumors in each experimental group were evaluated using a 10-parameter scoring system (Supplementary Table S10). The individual animal scores showed low within-group variability for several parameters; therefore, the histopathological findings were presented in a descriptive manner.

Considering the individual parameters, differences in histopathological scores were observed among the experimental groups (Figure 6). The vaccination regimens associated with the greatest tumor reduction (E5E7/E5E7, E5/E5E7, and E5/E7) generally exhibited low-to-moderate histopathological scores. In contrast, tumor histopathology showed a dissociation between the E7/E5 and E7/E5E7 groups. The E7/E5 group had a higher mean tumor size at the end of the experiment but comparatively lower histopathological scores, whereas the E7/E5E7 group showed marked tumor reduction together with higher scores for several histopathological parameters. Tumor cellular density (Figure 6A), necrosis (Figure 6B), and angiogenesis (Figure 6I) showed higher scores in the E7/E5E7 group, which also exhibited the highest inflammatory infiltration score (Figure 6E), whereas the other groups showed no variation.

Edema scores were lower in the vaccinated groups than in the control groups. Mitotic activity (Figure 6F) and cellular/nuclear pleomorphism (Figure 6G,H) showed similar patterns, with higher scores in the PBS groups and comparable scores among the vaccinated groups. Fibrosis (Figure 6C) showed lower scores in groups that received E5 as the priming immunization, whereas fibroblasts (Figure 6D) were lower in the E7/E5E7 and E5E7/E5E7 groups.

H&E-stained tumor sections from each experimental group are shown in Figure 7. Across the experimental groups, we observed areas of necrosis, fibrosis, edema, inflammatory infiltrates, and tumor-associated blood vessels. Representative sections from the E7/E5E7 group show increased inflammatory infiltrates and vascularization based on quantitative scoring, while sections from the E5E7/E5E7 group match the lower fibroblast and fibrosis scores seen in the histopathological analysis.

#### 2.8.2. Exploratory Immune Cell Profiling by Flow Cytometry

Flow cytometry immunophenotyping was performed on cryopreserved splenocytes, which were thawed and restimulated ex vivo with IL-2 and synthetic E5 and E7 peptides. The complete gating strategy is presented in Supplementary Figures S5–S8, while the statistical analyses are available in the Supplementary Tables S11–S14. Overall, the vaccination regimens exhibited distinct frequencies of the evaluated immune cell populations. Among the vaccinated groups, the E5/E5E7 and E5E7/E5E7 regimens showed the highest frequencies of CD4^+^ T cells, with both groups presenting significantly higher proportions than several of the other vaccination regimens (Figure 8A).

As regards CD8^+^ T cells, the E5/E7 and E5E7/E5E7 groups had significantly higher frequencies, with the E5/E7 regimen having a significantly higher frequency than the E7/E5, E5/E5E7, and E7/E5E7 groups (Figure 8B). Furthermore, CD80^+^ cell frequencies were significantly higher in all vaccinated groups compared to the pVAX control group, and among the vaccination regimens, the E5E7/E5E7 group exhibited the highest frequency, although this difference was not statistically significant when compared to the E7/E5 group (Figure 8C). Regarding CD206^+^ cells, the E5E7/E5E7 group had a significantly higher frequency than the E7/E5, E5/E5E7, and E7/E5E7 groups, but not significantly different from the E5/E7 group (Figure 8D). Finally, there was a difference in the profile of the cells in the different populations between the PBS and pVAX control groups, with the PBS group generally showing higher frequencies of the evaluated cell populations, while the pVAX group recorded relatively lower frequencies for several populations.

#### 2.8.3. Nitric Oxide Production

Nitric oxide production, measured by the Griess reaction, was significantly lower in the vaccination regimens associated with the greatest tumor protection (Figure 9). In contrast, PBS and pVAX exhibited the highest nitrite concentrations. Because nitric oxide levels visually paralleled the tumor growth profile, Pearson’s correlation analysis was performed using individual animal values (*n* = 33). The nitrite concentration showed a positive association between NO levels and tumor volume (r = 0.83; 95% CI: 0.68–0.91; *p* < 0.001) (Table S16). However, the E7/E5 group exhibited low NO levels (mean of 0.87 µM) despite a relatively high tumor volume (mean of 359.4 mm^3^), representing an exception to the general trend.

## 3. Discussion

Historically, the field of immunotherapy focused on the development of therapeutic vaccines against HPV has concentrated its efforts on targeting E6 and E7 [19,38,39]. Although DNA vaccines targeting these antigens have demonstrated efficacy in preclinical models, their translation into clinical trials has yielded limited results [40,41,42]. Furthermore, targeting these oncoproteins alone may not be sufficient to completely eliminate tumor cells. The expression of E6 and E7 is closely associated with HPV genomic integration, an event characteristic of the more advanced stages of the disease, such as high-grade lesions and invasive carcinoma [43,44]. In this context, vaccines targeting E6 and E7 exclusively may fail to reach infected basal cells that do not express these antigens or express them only minimally, which limits their efficacy in the early stages of infection [19].

The E5 protein, in turn, has been identified as a promising immunotherapeutic target, with a more apparent role in assisting the carcinogenic process and promoting immune evasion in cervical cancers and other HPV-associated neoplasms [16,30,45,46]. Although it is a small antigen with a hydrophobic structure, preclinical studies using the full-length protein or multi-epitope formulations have shown immunological efficacy by inducing specific CD4^+^ and CD8^+^ T-cell responses [30,47,48,49]. Furthermore, because E5 expression precedes E6 and E7 during the viral infection cycle and occurs predominantly in the early stages of carcinogenesis, it is particularly relevant to therapeutic treatments targeting precursor lesions [30,50]. The immunogenicity of these antigens can be further enhanced in DNA vaccines through the use of adjuvants, whether they are immunomodulatory molecules, delivery systems, or fusion strategies with adjuvant proteins [26,28,29,51]. In this regard, Paolini et al. (2017) [30] reported that DNA vaccines encoding E5 fused with the capsid protein of PVX had increased efficacy, with tumor regression rates of up to 80% in C3-Luc mouse models using homologous immunization regimens.

Therefore, the present study expands upon the previously proposed strategy for therapeutic DNA vaccines by combining immunoinformatics analyses, heterologous antigen regimens, and the incorporation of E5 and E7 coding sequences into a bicistronic plasmid. The immunoinformatics analyses provided a preliminary computational framework for evaluating the physicochemical and predicted immunological properties of the fused antigens and for guiding the design of the bicistronic construct. However, these predictions were not intended to establish functional expression or predict in vivo therapeutic efficacy. At the computational level, the full-length constructs were predicted to be non-toxic and to retain several relevant epitopes after fusion with CP. An important observation from the MHC-II predictions was that fusion of E5 to CP altered the predicted helper epitope repertoire. Although several E5-derived binding cores were conserved, some native sequence contexts were replaced by CP-derived peptides. Therefore, the CD4^+^ helper activity potentially associated with E5CP cannot be attributed exclusively to native E5-derived determinants and may also be influenced by the fused CP sequence. Considering that C-IMMSIM still does not support murine MHC alleles, in silico immunogenicity analyses were conducted within a human HLA context. In this scenario, more pronounced responses were observed for HaE7CP, whereas the bicistronic vaccine and HisE5CP elicited intermediate and low responses, respectively. This simulation is therefore a general, hypothesis-generating immunogenicity sketch and not a model of the C57BL/6 mice. Consistent with this limitation, the in vivo studies revealed more complex dynamics among the vaccination groups, with regimens initiated with E5 consistently yielding greater therapeutic efficacy.

The in vivo data showed that the majority of the regimens tested were associated with a lower tumor volume and also suggested that the order of administration of the antigens may influence the antitumor response. Vaccines initiated with E5, with or without E7, tended to show greater tumor volume reduction. A similar pattern was observed when E5 and E7 were co-encoded in the bicistronic construct, although its effect was not statistically distinguishable from the combination of monocistronic E5 and E7. Specifically, the homologous bicistronic E5E7/E5E7 regimen resulted in a 69.1% reduction compared to the PBS group, followed by the heterologous regimens E5/E5E7 (60.9%) and E5/E7 (57.0%). In contrast, the E7/E5 regimen did not significantly reduce tumor growth, suggesting that in this context, priming with E5 may contribute to therapeutic efficacy. This finding is consistent with the role of E5 in the early stages of infection and with its ability to induce helper CD4^+^ T-cell responses, which may promote the expansion and functionality of CD8^+^ T-cell responses against E7 [30,46]. On the other hand, regimens that primarily used E7 as the first dose did not follow a uniform pattern. While E7/E5E7 promoted significant tumor reduction (53.7%), E7/E5 showed no efficacy, indicating that the presence of E7 in the booster dose can, at least partially, restore the immune response when priming is performed with E7.

Different histopathological profiles were observed in the E7/E5 and E7/E5E7 groups despite the differences in tumor volume. The E7/E5 group showed comparatively lower histopathological scores despite having larger-volume tumors at sacrifice, with a mean tumor volume of 359.4 mm^3^ and relatively low scores for necrosis, inflammatory infiltrate, and angiogenesis. The E7/E5E7 group showed higher scores for several parameters related to tissue remodeling, including angiogenesis, fibrosis, tumor cellular density, necrosis, and inflammatory infiltrate, despite the marked tumor reduction. Although there are few detailed histopathological studies in HPV vaccination models, immune cell-mediated destruction may be accompanied by temporary tissue changes associated with inflammation and tissue remodeling, including tumor necrosis, immune cell infiltration, and neovascularization [52,53].

Based on flow cytometry results, higher frequencies of immune cell subpopulations were observed in splenocytes from the PBS-treated group. This phenomenon may be due to a lack of antigen-specific immune activation, which keeps naive and central memory T cells in secondary lymphoid tissue and reduces their recruitment to the tumor site [52,54,55]. In the case of antigen-specific vaccination, one would expect the expansion of effector T cells and their migration to the tumor microenvironment, where their antitumor activities are expected to manifest [56,57]. This result means that low frequencies in the vaccinated group may indicate immune cell trafficking rather than the absence of immune activation. Furthermore, pVAX consistently showed lower frequencies of CD4^+^, CD8^+^, and CD80^+^ cells than those observed in the PBS and vaccinated groups. Although pVAX is a plasmid containing immunostimulatory motifs, it did not express an HPV antigen; therefore, it elicited only a transient, nonspecific immune response.

Production of nitric oxide as measured by the Griess assay in the supernatants of restimulated splenocytes correlated positively with tumor size (r = 0.83, *n* = 33, 95% CI 0.68–0.91). Groups exposed to immunization regimens that resulted in therapeutic effects had the lowest NO levels, although the E7/E5 group represented an exception to the overall pattern, displaying a relatively large mean tumor volume despite low NO detection. NO is a versatile messenger whose effects on the tumor process are determined by its concentration, cell type of origin, and inflammation. Although transient NO production is involved in the immune response against tumors, sustained NO generation leads to immunosuppression and suppression of T-cell activity, which are associated with tumor growth [58,59,60,61]. Overall, the observed association between NO production and tumor volume should be considered exploratory and hypothesis-generating. Additional studies are required before considering NO as a potential marker associated with tumor progression in this model.

Furthermore, the lack of functional cellular immunity assays, such as ELISPOT, limits the characterization of the induced immune responses. Although flow cytometry provided exploratory information that, when combined with tumor reduction, correlation with nitric oxide levels, and histopathological findings, allowed us to infer the activation of relevant immunological pathways, the functionality of these cells remains unassessed. Future studies should incorporate intracellular cytokine flow cytometry and cytotoxicity assays to confirm the functional profile of T cells induced by the different immunization strategies. The present study was also conducted exclusively in female mice, in line with the preclinical evaluation of these vaccination strategies. Although this experimental design is appropriate for the disease model under investigation, future studies including male animals will also be important to determine whether the observed responses apply to both sexes. Another limitation is that all vaccination regimens were evaluated using 50 µg of DNA, which corresponds to half the dose previously reported for homologous vaccination with E5CP. Tumor volume was measured by caliper without blinding to group allocation, which should be considered when interpreting the antitumor efficacy results. In addition, splenocytes were cryopreserved for six months before analysis, which can bias cell recovery and viability, and CD206 alone is a weak marker for an M2 phenotype.

Regarding protein-level expression, the HPV16 E5 protein is a small, hydrophobic polypeptide of ~9–10 kDa, while the Potato Virus X CP is ~25 kDa. As predicted by ProtParam and taking into account the addition of the histidine tag, the molecular weight of the chimeric protein is ~36 kDa. Due to strong self-interaction and hydrophobic associations, the E5 protein displays anomalous migration patterns in Western blot analyses [62,63]. Wetherill et al. (2012) [63] showed that GFP-fused E5 protein in transfected HEK293 cells appeared as multiple bands, including dimers (~20 kDa) and higher-order multimeric structures (~64 to 148 kDa), similar to the sizes observed in our results (Figure S4). For E7CP, chemiluminescent detection showed several bands, including those around the predicted molecular weight, in both monocistronic and bicistronic conditions. However, similar bands were detected in the empty vector control, leading to substantial background and preventing us from unambiguously assigning those bands to E7CP. Therefore, conventional PCR detection of transcripts should be interpreted as evidence of transcription, rather than definitive proof of protein expression, and future studies using complementary protein-level approaches will be necessary to confirm co-expression of both proteins from the bicistronic construct. In addition, investigating tumor expression patterns by RT-qPCR and immunohistochemistry using cellular markers will be important to understand the mechanisms underlying the differential efficacy among the regimens.

## 4. Methodology

### 4.1. Vaccine Constructs and Study Design

In this study, we designed a new bicistronic DNA vaccine construct (pVAX-HisE5CP–IRES–HaE7CP) containing the coding sequences of E5CP and E7CP separated by an internal ribosome entry site for use in heterologous prime–boost immunization strategies. The monocistronic DNA vaccines pVAX-E5CP and pVAX-E7CP were previously described and experimentally validated in vivo using homologous immunization regimens [30]. Although the therapeutic efficacy of these monocistronic vaccines has been previously demonstrated, their physicochemical, structural, and immunological properties have not been systematically characterized using computational approaches. Therefore, in silico analyses were performed to support the rational design of the bicistronic construct and provide a comparative framework for interpreting subsequent findings from the different vaccination strategies.

### 4.2. In Silico Characterization of E5CP, E7CP, and Bicistronic Constructs

#### 4.2.1. In Silico Prediction of Physicochemical Properties, Toxicity, and Allergenicity

The physicochemical properties of the chimeric proteins HisE5CP and HaE7CP were calculated using the ProtParam tool [31]. The evaluation of potential toxicity was conducted using the ToxinPred server [64], and the prediction of allergenic potential was performed using AllerTOP v.2.0 [65].

#### 4.2.2. RNA Secondary Structure Prediction

The secondary structures of the mRNA transcripts for HisE5CP, HaE7CP, and the bicistronic pVAX-HisE5CP–IRES–HaE7CP constructs were predicted using the RNAfold web server (http://rna.tbi.univie.ac.at/ (accessed on 16 July 2026)) [66]. Predictions were performed at default parameters. For each sequence, the minimum free energy (MFE) structure, ensemble free energy, base-pairing probabilities, and structural diversity were calculated. Both the MFE and centroid structures were generated for comparison. The predicted structures were visualized and exported directly from the RNAfold output.

#### 4.2.3. Prediction of Three-Dimensional Structures and Structural Quality Assessment

To predict the 3D structures of the chimeric proteins, we used the AlphaFold3 Server [32]. After structural prediction, the constructions were evaluated in ModFold8 to check the overall quality of the models [33] and were analyzed for atom contacts, conformational stability, and possible rotamers using the MolProbity web service v4.5.2 [34]. In addition, we refined the original AlphaFold3 file using the ReFOLD3 server [35] to investigate whether we could further improve model quality.

#### 4.2.4. Preservation of Immunologically Relevant Epitopes Following Fusion to CP

To verify the post-processing preservation of the most immunologically relevant E5 and E7 epitopes after fusion with CP, epitope prediction was performed based on the protein sequence of the antigens versus the reference sequences of E5 (NP_041330.2) and E7 (NP_041326.1). Epitopes were predicted using the online server Immune Epitope Database and Analysis Resource (IEDB) (http://www.iedb.org/ (accessed on 13 June 2026)). For MHC-I, the classic H-2-Db and H-2-Kb allele sets of C57BL/6 were used. In this context, the epitope sequence length (9–10 amino acids), percentile classification <1, and immunogenicity score > 0.5 were considered. For MHC class II prediction, the H2-IAb allele was selected, and predicted peptides were ranked according to the NetMHCIIpan EL percentile rank.

#### 4.2.5. Immunological Simulation Using the C-IMMSIM Server

Immunological simulation was performed on the C-IMMSIM server (https://kraken.iac.rm.cnr.it/C-IMMSIM/ (accessed on 1 September 2025)) to characterize the immune response profiles and immunogenicity of the monocistronic and bicistronic vaccines. The entire simulation was performed in 75 time steps, corresponding to approximately 25 days. Two injections of the immunogens were administered one week apart at time steps 9 (day 3) and 33 (day 11), and the adjuvant value was set at 100. To achieve comprehensive population coverage, the HLA alleles A0201, A0301, B0702, B0801, DRB10301, and DRB11501 were selected. Because C-IMMSIM simulations were performed using human HLA alleles, these predictions were not intended to model the H-2b immune context of the C57BL/6 mice used in the in vivo experiments. Instead, they were used exclusively as a general qualitative assessment of the predicted immunogenic profiles of the vaccine constructs.

### 4.3. Plasmid Construction and Cloning

#### Design and Construction of the Bicistronic DNA Vaccine

To generate the bicistronic construct, codon-optimized sequences encoding E5CP and E7CP were designed for *Homo sapiens* expression and assembled into the pcDNA3.1 backbone, separated by an ECMV-IRES (Internal Ribosome Entry Site of Encephalomyocarditis virus) synthesized by GenOne Biotechnologies (Rio de Janeiro, Brazil). This strategy was applied to ensure sequence integrity and facilitate subsequent subcloning into the pVAX1 vector. The assembled bicistronic cassette was subcloned into the pVAX1 vector using *Bam*HI and *Xba*I restriction enzymes, resulting in the pVAX-HisE5CP–IRES–HaE7CP construct. Correct insertion and the expected plasmid architecture were confirmed by restriction analysis. Endotoxin-free plasmid DNA was prepared using a GenElute HP Endotoxin-Free Plasmid DNA Megaprep Kit (Sigma-Aldrich, St. Louis, MO, USA) for subsequent in vitro and in vivo experiments.

### 4.4. In Vitro Expression of Vaccine Constructs

#### 4.4.1. Cell Culture and Transient Transfection

Human embryonic kidney cells (HEK-293T), provided by Dr. Lucas Matheus Barreto Santana from the Aggeu Magalhães Institute (IAM—Fiocruz, Recife, Brazil), were cultured in Dulbecco’s Modified Eagle Medium (DMEM; Gibco, Waltham, MA, USA) with 10% fetal bovine serum (FBS), 100 U/mL penicillin, and 100 μg/mL streptomycin, and maintained at 37 °C in a humidified 5% CO_2_ atmosphere. For transfection assays, cells were seeded in 6-well plates at a density of 3 × 10^5^ cells per well and allowed to reach approximately 70–80% confluence. Cells were transiently transfected in triplicate with the plasmids pVAX-HisE5CP, pVAX-HaE7CP, pVAX-HisE5CP-IRES-HaE7CP, or the empty vector pVAX1, using Lipofectamine™ 3000 (Thermo Fisher Scientific, Waltham, MA, USA), according to the manufacturer’s instructions. After transfection, cells were incubated for 48 h prior to RNA extraction.

#### 4.4.2. Qualitative Analysis of Transcript Expression by PCR from cDNA

Total RNA was extracted from transfected cells using TRIzol™ reagent (Invitrogen™, Carlsbad, CA, USA), following the manufacturer’s protocol. RNA samples were treated with DNase I and subsequently reverse-transcribed into cDNA using the Maxima First Strand cDNA Synthesis Kit (Thermo Scientific). Conventional PCR was performed using cDNA as the template. Primer pairs specific to E5, E7, and IRES-E7 sequences (Supplementary Table S1) were used to qualitatively confirm transcription of the vaccine constructs following transfection. PCR products were resolved by agarose gel electrophoresis and visualized under UV illumination after SYBR™ Safe DNA Gel Stain (Invitrogen) staining.

#### 4.4.3. Protein Expression Analysis by Western Blot

For protein expression analysis, and considering the predicted cysteine-rich regions and moderate hydrophobicity of the chimeric proteins, transfected HEK-293T cells were harvested 24 h post-transfection and lysed in RIPA buffer (Sigma-Aldrich) supplemented with PMSF protease inhibitor. Protein precipitation was performed using trichloroacetic acid and cold acetone with 50 mM DTT, and the pellet was resuspended in 120 µL of 2X Laemmli sample buffer. Following this, 20 µL of total protein was separated by SDS-PAGE and transferred to PVDF membranes. Membranes were blocked with 1X PBS with 1% casein (Bio-Rad) and incubated overnight at 4 °C with primary antibodies. Mouse anti-polyhistidine monoclonal antibody (Sigma-Aldrich) was used at 1:3000 to detect E5CP, and anti-HA antibody (Abcam, Cambridge, UK) was used at 1:5000 to detect E7CP. After washing, membranes were incubated with HRP-conjugated anti-mouse IgG secondary antibody (Sigma-Aldrich). His-tagged protein bands were visualized using NBT/BCIP for 5 min in the absence of light. HA-tag was detected by chemiluminescence using the ECL Western Blotting Substrate (Thermo Fisher) and image acquisition with the C-DiGit Blot Scanner (LI-COR, St. Lincoln, NE, USA), using Image Studio Software (v.5.2).

### 4.5. In Vivo Trials

#### 4.5.1. Characteristics of the Animal Model and Quality Controls

The in vivo experiments were carried out at the Department of Genetics of the Federal University of Pernambuco, which holds Biosafety Quality Certificate No. 0158/02.0. A total of 35 specific pathogen free (SPF) female C57BL/6 mice, aged 4–7 weeks, were obtained from the Animal Facility Center (Centro de Bioterismo—CEBIO) of the Federal University of Pernambuco and housed in the animal facility of the Keizo Asami Institute (iLIKA) under controlled environmental conditions (temperature 22 ± 2 °C, relative humidity 55 ± 10%, 12 h light/dark cycle). The animals were fed a standard rodent diet with water ad libitum and were monitored periodically for tumor growth and signs of pain, distress, or significant weight loss. Once tumors measuring 2 cm in diameter or width were detected in any experimental group, euthanasia was performed via cervical dislocation following anesthesia with a mixture of ketamine (300 mg/kg, 10%) and xylazine (30 mg/kg, 2%), and the experiment’s endpoint was established. All procedures were approved by the Animal Ethics Committee (Comissão de Ética no Uso de Animais—CEUA) of the Federal University of Pernambuco (Process No. 0068/2022, approved ad referendum on 28 January 2025, with validity from 2 October 2022 to 2 March 2026) and were conducted in accordance with the guidelines of the Brazilian National Council for the Control of Animal Experimentation (CONCEA).

#### 4.5.2. Tumor Challenge and Vaccination Protocol

Female C57BL/6 mice were subcutaneously inoculated with 5 × 10^5^ C3 tumor cells (HPV16 complete genome) suspended in 30 μL of PBS, as previously described [47]. On the day of tumor inoculation, 35 animals were randomly assigned to seven experimental groups (*n* = 5 per group), including homologous and heterologous antigen vaccination regimens and two control groups (PBS and empty pVAX1). The vaccines were given intramuscularly (50 μg of plasmid DNA in 30 μL of saline solution) into the left *tibialis* muscle, followed by in vivo electroporation with two 100 V pulses for 20 ms, using BTX Harvard Apparatus equipment. Immunizations were performed on days 3 and 10 post-tumor inoculation. Tumor growth was monitored periodically using digital calipers, and volume was calculated using the formula (width^2^ × length × 0.52). At the experimental endpoint, tumors and spleens were collected for histological and immunological analyses.

### 4.6. Immunological and Histological Analyses

#### 4.6.1. Histopathological Analysis

Excised tumors were fixed in 10% neutral buffered formalin for 24 h, processed by routine histological procedures in the Cytological and Molecular Research Laboratory (LPCM), embedded in paraffin, and sectioned at a thickness of 8 μm. For each tumor, one transverse and one longitudinal section were evaluated; in some cases, additional sections were prepared when repetition was necessary. Tissue sections were stained with hematoxylin and eosin (H&E) and examined using a Nikon^®^ (Walt Whitman Rd, Melville, NY, USA)light microscope coupled to Motic Images Plus 3.0 software (Motic, Universal City, TX, USA). Histopathological evaluation was performed in a blinded manner by assessing tumor cell density, necrotic area, fibrotic area, inflammatory infiltrate, mitotic activity, cellular and nuclear pleomorphism, angiogenesis, fibroblast presence, and edema. Each histopathological parameter was scored individually for each animal according to lesion intensity as absent (0), mild (1), moderate (2), or severe (3).

#### 4.6.2. Splenocyte Isolation and Ex Vivo Stimulation

Following euthanasia, spleens were aseptically collected and processed as previously described [67]. Splenic tissue was mechanically dissociated, and erythrocytes were removed by hypotonic lysis. Cell suspensions were cryopreserved in freezing medium containing 90% fetal bovine serum and 10% DMSO and stored at −80 °C. After six months, splenocytes were thawed and washed, and mononuclear cells were isolated by density gradient centrifugation using Ficoll-Paque PLUS (1.077 g/mL). Cryopreserved samples from all experimental groups were analyzed simultaneously to minimize inter-assay variability. Cells were plated at a density of 1 × 10^5^ cells per well in 48-well plates and initially stimulated with recombinant IL-2 (10 IU/mL) to support cell viability [68,69]. After 24 h, cultures were restimulated with 5 μg/mL of synthetic E5 (VCLLIRPLL) and E7 peptides (RAHYNIVTF) and were maintained at 37 °C in a humidified 5% CO_2_ atmosphere. Cells and supernatants were subsequently collected for immunological analyses.

#### 4.6.3. Immunophenotyping

Phenotypic characterization of immune cell populations was performed by flow cytometry using a BD Accuri™ C6 Plus cytometer. Restimulated splenocytes were stained with fluorochrome-conjugated monoclonal antibodies specific for mouse CD4 (APC), CD8b (FITC), CD80 (PE), CD14 (APC), and CD206 (FITC). A minimum of 7000 events per sample was acquired and analyzed.

#### 4.6.4. Nitric Oxide (NO) Measurement

The concentrations of NO_2_^−^ in splenocyte culture supernatants were measured using the Griess reaction [70,71,72]. A total of 100 μL of each supernatant was incubated with 100 μL of Griess reagent at room temperature for 10 min. The NO_2_^−^ concentration was measured by reading the optical density at 550 nm (A550) with a GloMax^®^ Discover Microplate Reader and comparing these values to the NaNO_2_ standard curve (100; 50; 25; 12.5; 6.25; 3.13; 1.56 and 0 µM).

### 4.7. Statistical Analysis

Statistical analyses were performed using version 9.0.0 of GraphPad Prism software (GraphPad Software, San Diego, CA, USA). Data normality was evaluated using the Shapiro–Wilk test. Tumor volume, CD80^+^, CD4^+^, and CD8^+^ data passed the normality test in all groups (*p* > 0.05). For nitric oxide and CD206^+^, six of the seven groups passed the normality test, with a deviation in the E5/E5E7 group for NO (W = 0.77, *p* = 0.047) and in the E7/E5E7 group for CD206^+^ (W = 0.66, *p* = 0.0037). The Brown–Forsythe test supported the homogeneity of variances for all variables (*p* > 0.05). Given the homogeneity of variances and the robustness of one-way ANOVA to mild normality deviations, parametric analysis was retained: one-way ANOVA followed by Dunnett’s multiple comparison test for tumor volume (comparisons against the PBS group) and one-way ANOVA followed by Tukey’s post hoc test for nitric oxide and flow cytometry data. The ANOVA model explained 85.6% of the variance in nitric oxide levels (R^2^ = 0.8563) and 85.9% of the variance in CD206^+^ frequencies (R^2^ = 0.8589); however, due to the small sample size (*n* = 5 per group, except for pVAX with *n* = 3), this study may be underpowered to detect small effect sizes. Correlation between nitric oxide levels and tumor volume was determined using Pearson’s correlation coefficient. Differences were considered statistically significant at *p* < 0.05 (* *p* < 0.05, ** *p* < 0.01, *** *p* < 0.001, and **** *p* < 0.0001).

## 5. Conclusions

This study expands upon the therapeutic vaccination strategy that was originally proposed by Paolini et al. by integrating in silico, in vitro, in vivo, and ex vivo assessments. Although the proposed strategy did not outperform homologous vaccination with four doses of E5CP, it expanded our understanding of how antigen combinations and the vaccination sequence influence therapeutic efficacy. Our results suggest that heterologous antigen regimens and bicistronic vectors containing HPV16 E5 and E7 sequences are associated with distinct tumor and exploratory immunological profiles depending on the antigenic composition and order of administration. Furthermore, a higher or lower prevalence of pro-inflammatory populations on its own did not necessarily indicate antitumor efficacy; for a more accurate overall assessment of the preclinical efficacy of a therapeutic vaccine, techniques that visualize different aspects of the immune response are also necessary. Parameters such as the assessment of tumor volume throughout the experiment, histopathological analyses, and nitric oxide measurements were essential for clarifying the mechanisms underlying the different regimens. However, it is important to emphasize that these techniques need to be even more specific to identify, with greater precision, the mechanistic factors by which some of these vaccines produced therapeutic effects. Furthermore, dose escalation, the use of alternative delivery systems, and combination with other forms of immunotherapy will be important for enhancing the therapeutic efficacy of these vaccine strategies.

## Figures and Tables

**Figure 1:**
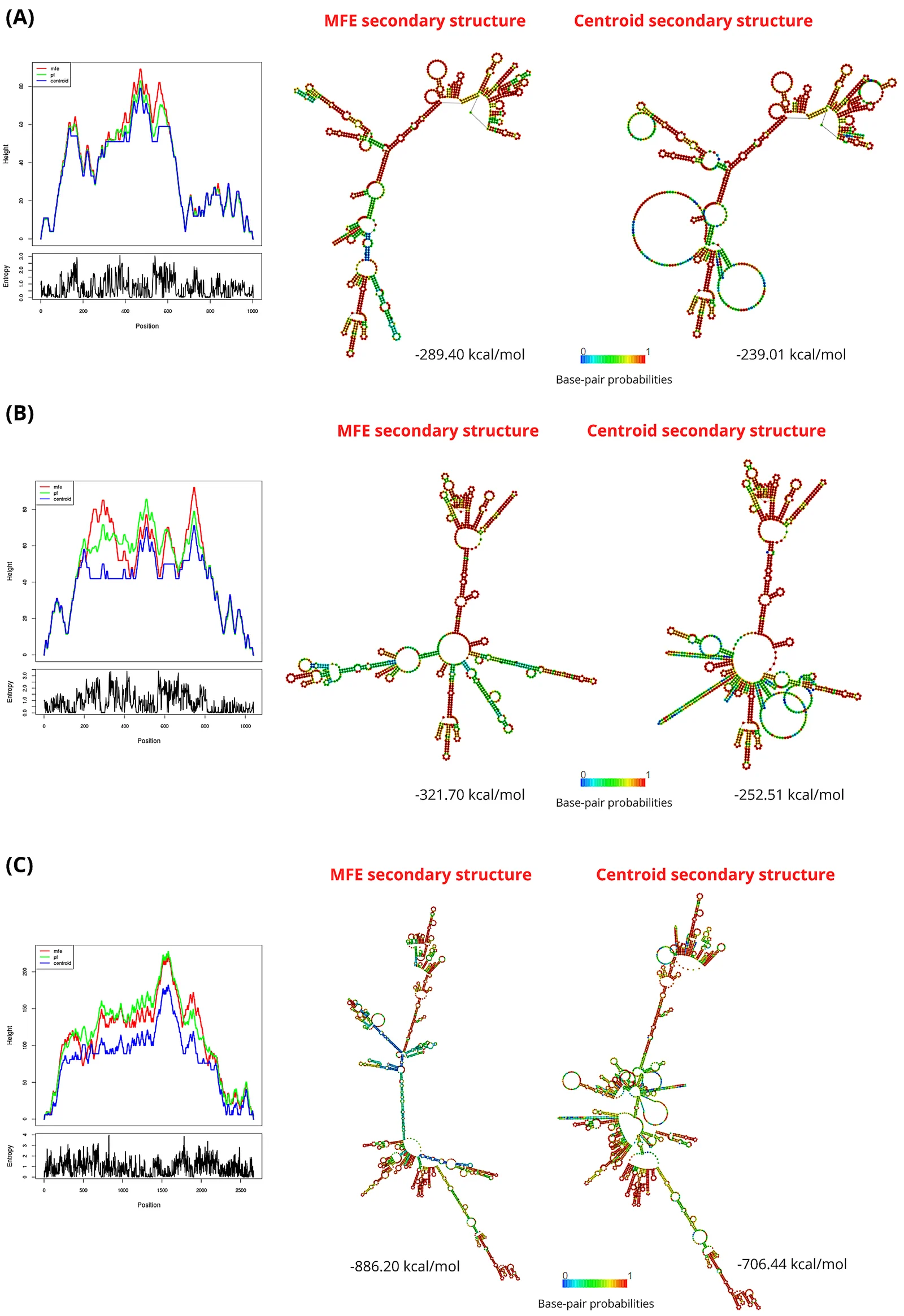
Secondary RNA structures of HisE5CP, HaE7CP, and the bicistronic construct predicted by RNAfold. The MFE, a mountain plot representation of the MFE structure, and the centroid secondary structure are illustrated in (**A**) HisE5CP, (**B**) HaE7CP, and (**C**) HisE5CP-IRES-HaE7CP. Results have been computed using RNAfold 2.6.3. In this figure, colors are applied to represent the probability of each base pair in all two-dimensional RNA structures.

**Figure 2:**
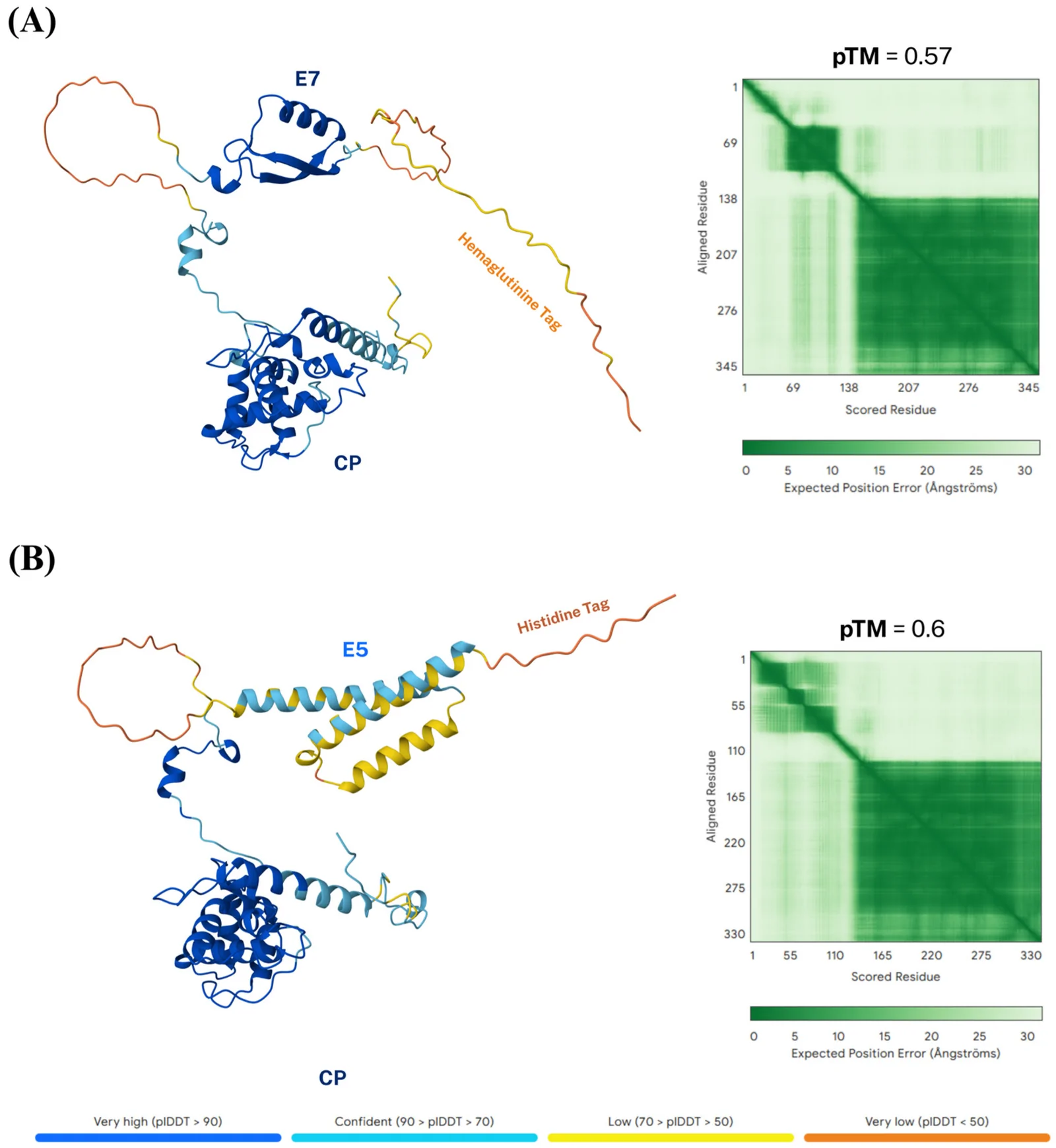
3D predictions of HisE5CP and HaE7CP. In (**A**), the predicted structure of the HaE7CP chimeric protein is shown, while in (**B**), the predicted structure of the HisE5CP, containing the E5 and CP proteins with a histidine tag, is shown. The expected positional error matrices are shown to the right of each structural model.

**Figure 3:**
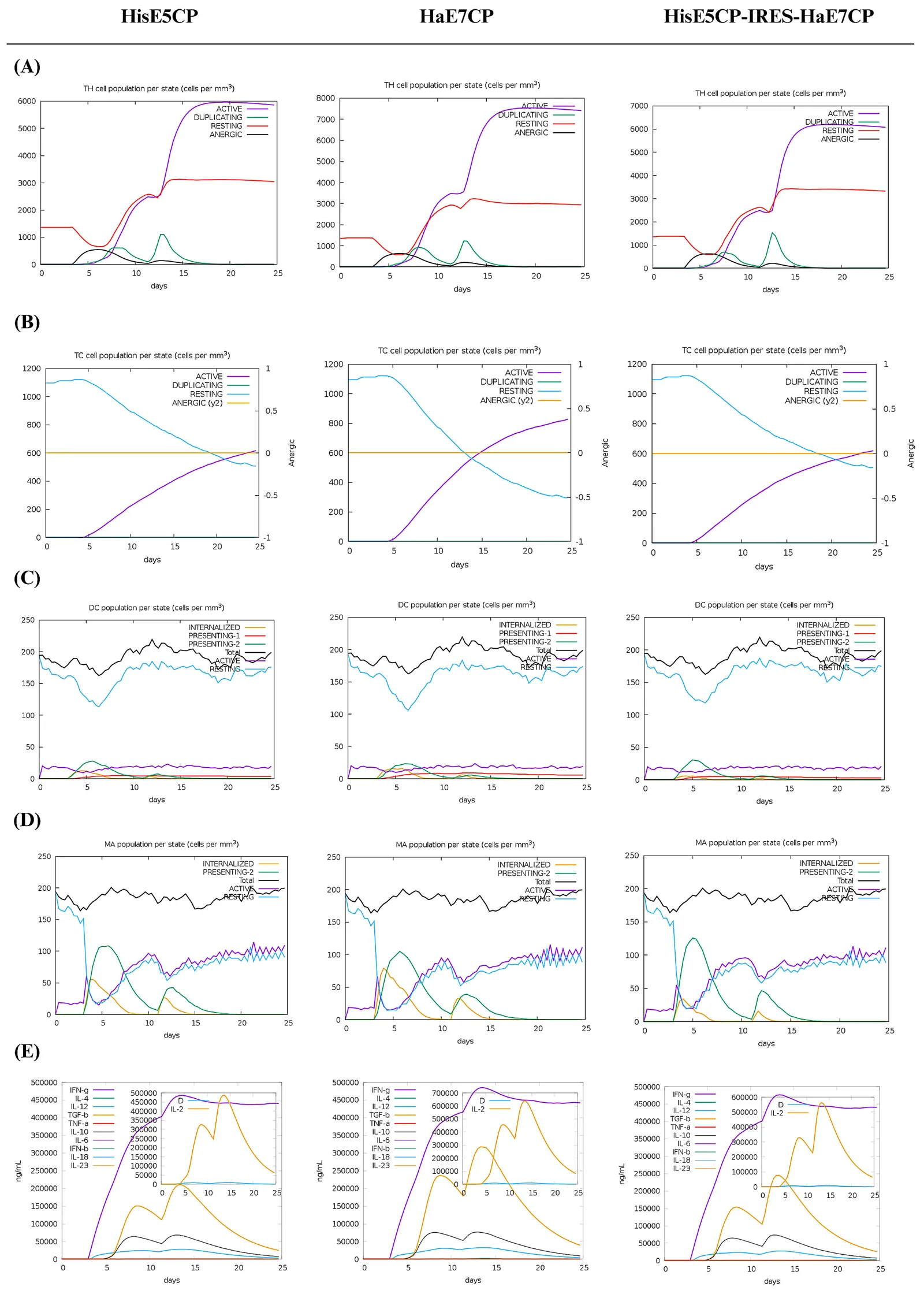
Cellular responses, dendritic cell and macrophage profiles, and cytokine secretion for constructs with E5 and E7 predicted by C-IMMSIM within a human HLA framework. (**A**) shows the T-helper cell population per state. In (**B**), the CD8 T population per entity state shows counts for active, duplicating, and anergic cells. (**C**,**D**) show the predicted response and activation for DCs and macrophages, and (**E**) the cytokine profile induced.

**Figure 4:**
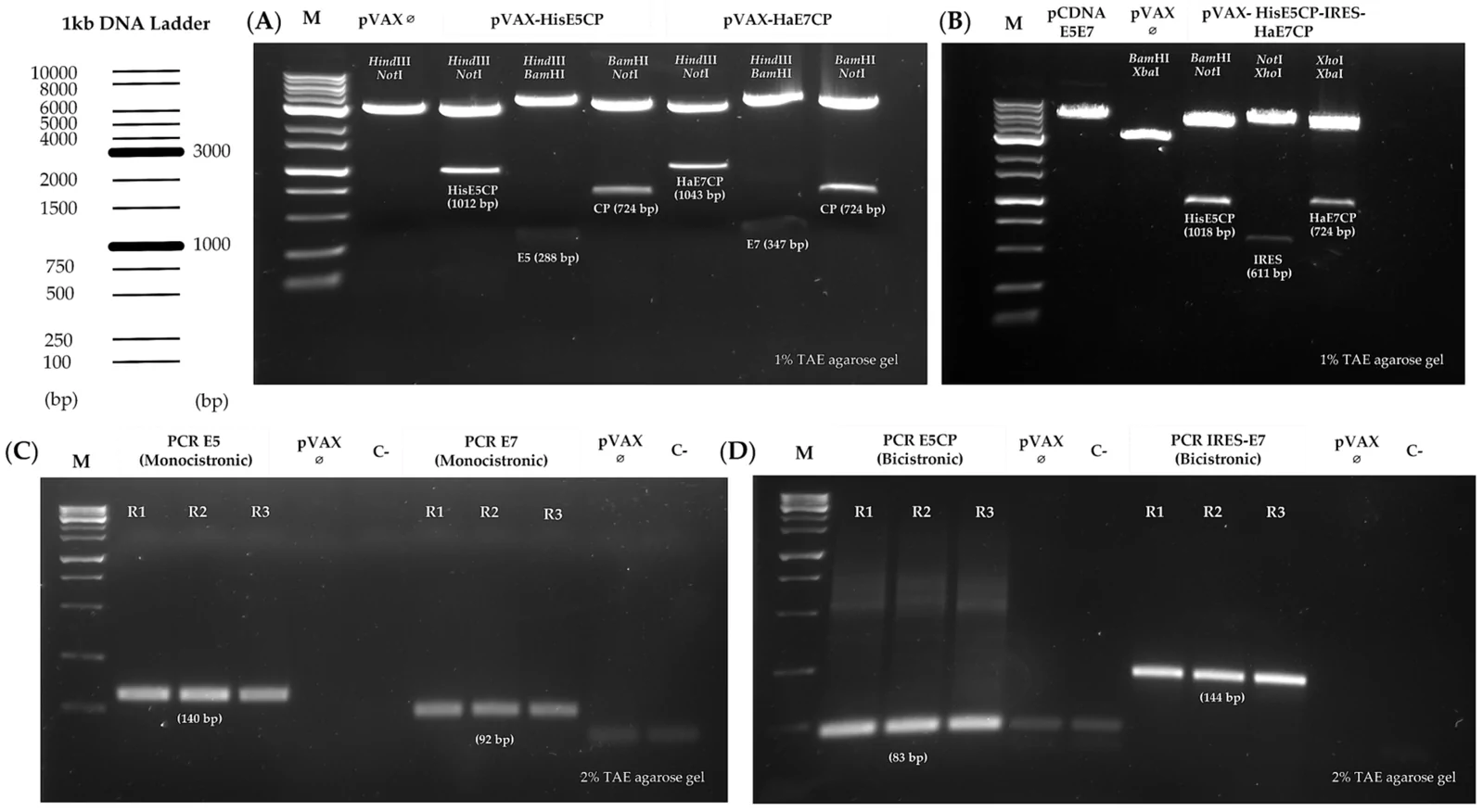
Restriction digestion of constructs and reverse transcription–conventional PCR analysis of the vaccine transcripts. (**A**,**B**) Digestion profiles of monocistronic (**A**) and bicistronic (**B**) vectors on 1% agarose gel. (**C**,**D**) RT-PCR of transcripts from transfected HEK-293T cells on 2% agarose gel. M: 1 kb DNA ladder (Kasvi, K9-1000L); C-: negative control; pVAX Ø: empty vector.

**Figure 5:**
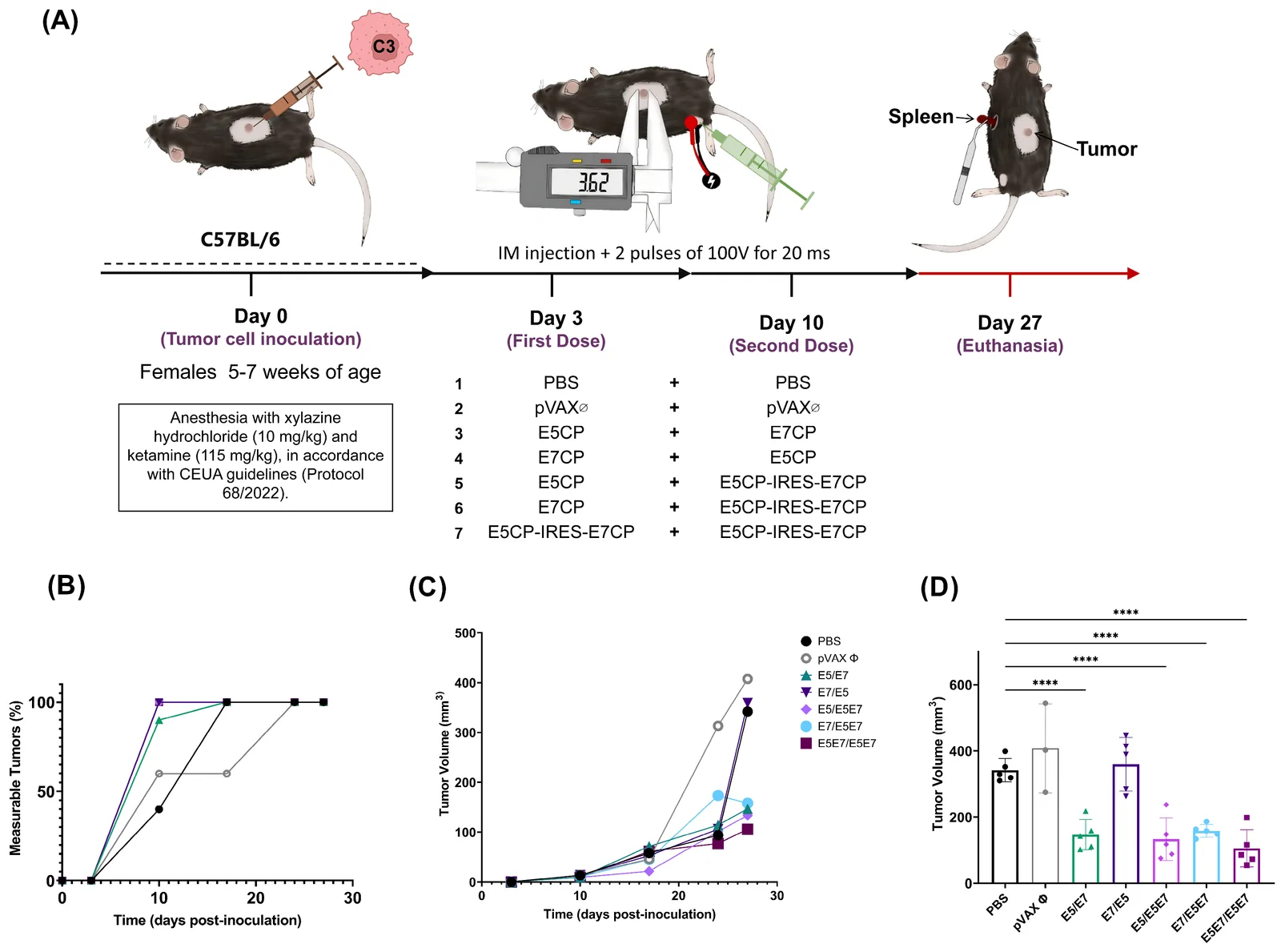
Therapeutic efficacy of the DNA vaccination regimens in the C3 HPV16 tumor model. (**A**) Experimental design. Female C57BL/6 mice (*n* = 5 per group) were inoculated subcutaneously with 5 × 10^5^ C3 cells on day 0 and vaccinated by intramuscular injection with 50 μg/dose followed by electroporation on days 3 and 10. Animals were euthanized on day 27 for tumor and spleen collection. (**B**) Percentage of mice bearing measurable tumors during follow-up. (**C**) Tumor growth curves throughout the experiment. (**D**) Tumor volume at the experimental endpoint. Statistical significance was determined by one-way ANOVA followed by Dunnett’s multiple comparison test. Only comparisons with the PBS group are shown for clarity. **** *p* < 0.0001. No statistically significant difference was observed between the bicistronic E5E7/E5E7 regimen and the monocistronic combinations E5/E5E7, E7/E5E7, or E5/E7 (*p* > 0.05).

**Figure 6:**
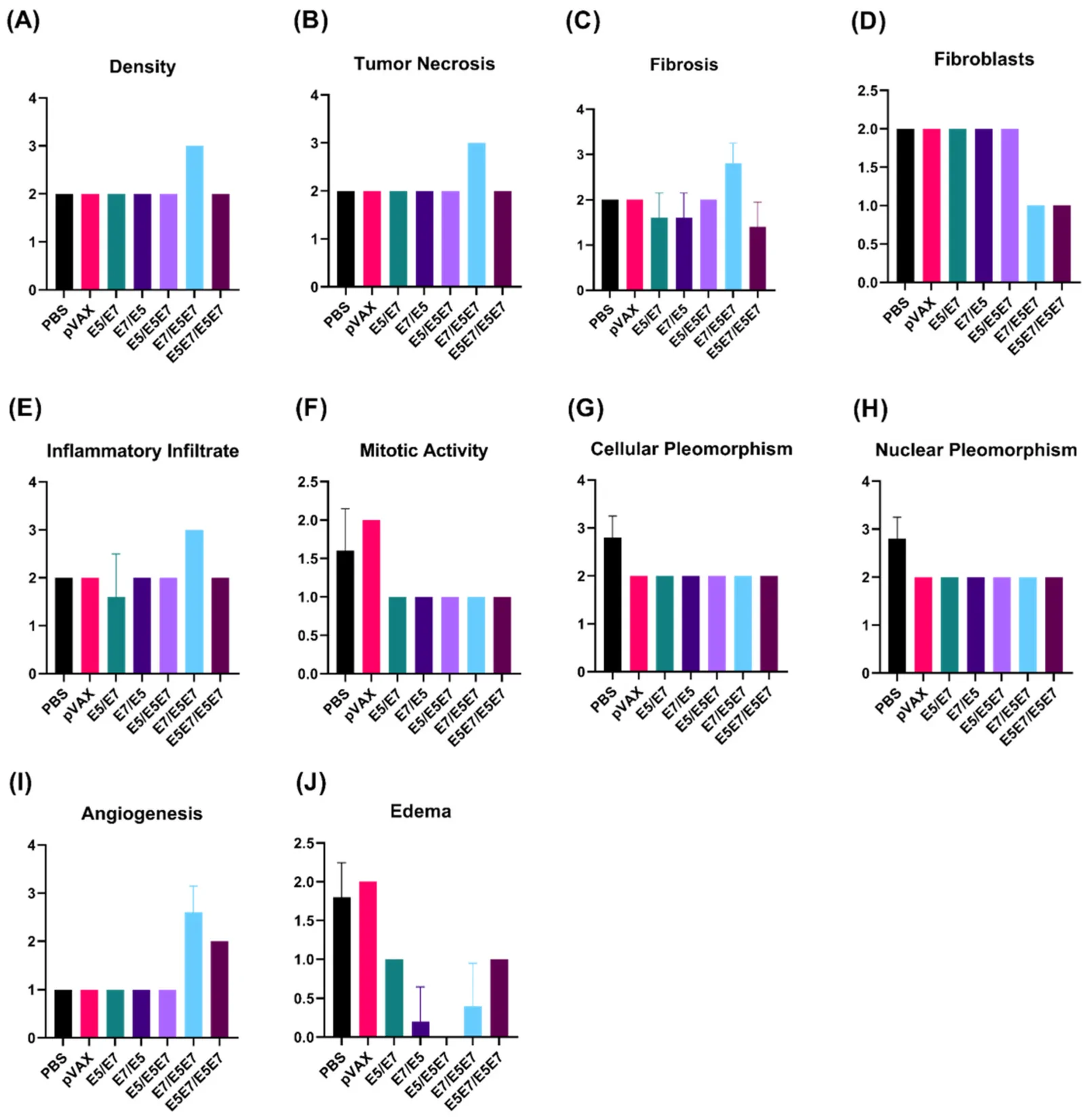
Histopathological analysis of tumors from each experimental group. (**A**) Tumor cellular density; (**B**) Area of necrosis; (**C**) Area of fibrosis; (**D**) Fibroblasts; (**E**) Inflammatory infiltrate; (**F**) Mitotic activity; (**G**) Cellular pleomorphism; (**H**) Nuclear pleomorphism; (**I**) Tumor angiogenesis; and (**J**) Edema.

**Figure 7:**
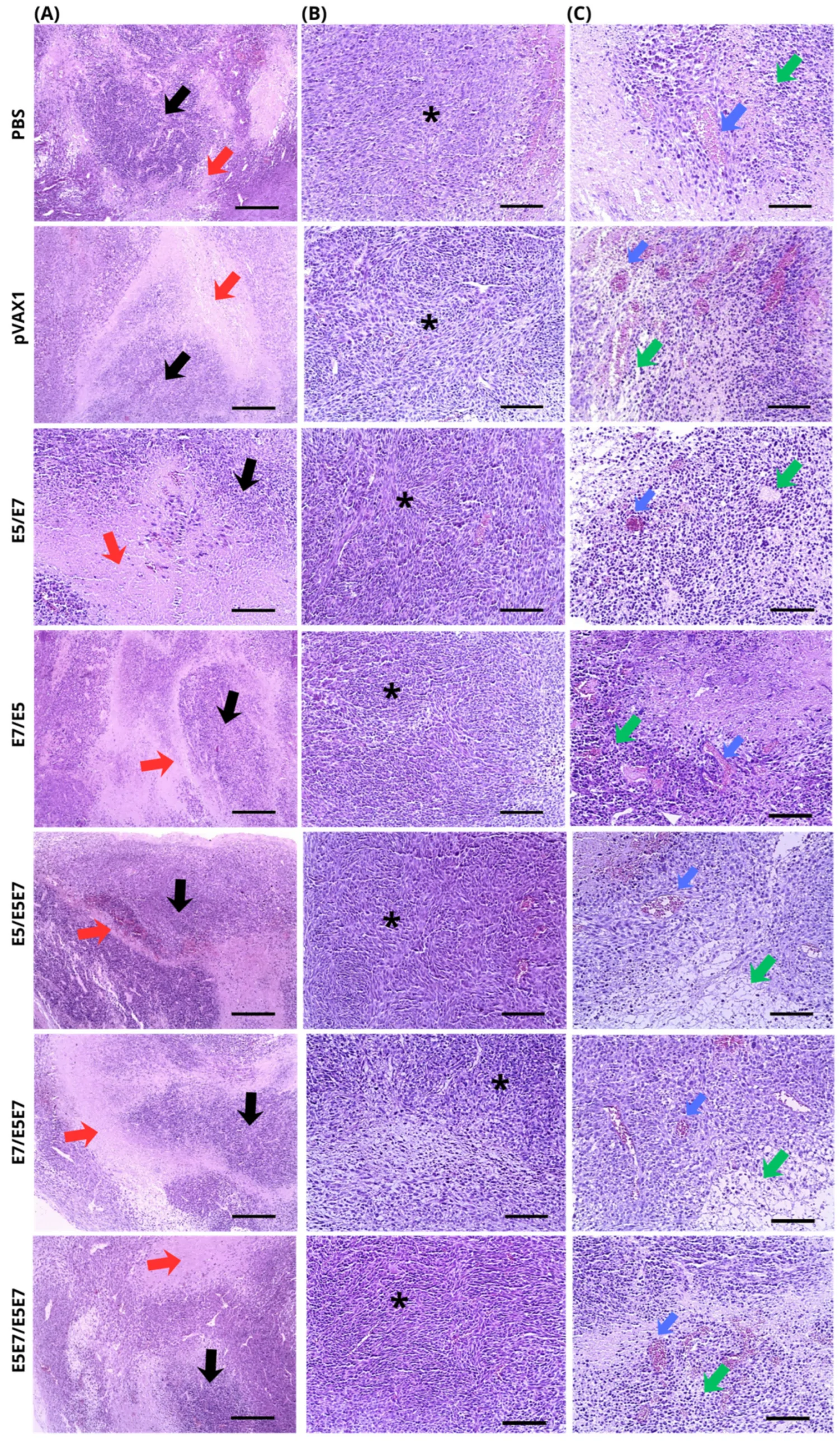
Representative histopathological sections of tumors from each experimental group. Representative H&E-stained tumor sections illustrating the main histopathological features observed in each vaccination regimen. (**A**) The left column (40×) highlights areas of tumor necrosis (black arrows) and fibrosis (red arrows). (**B**) The middle column (100×) shows representative tumor morphology, including tumor cellularity and pleomorphism (asterisks). (**C**) The right column (100×) illustrates inflammatory infiltrates (green arrows) and tumor-associated blood vessels (blue arrows). Scale bar = 100 μm.

**Figure 8:**
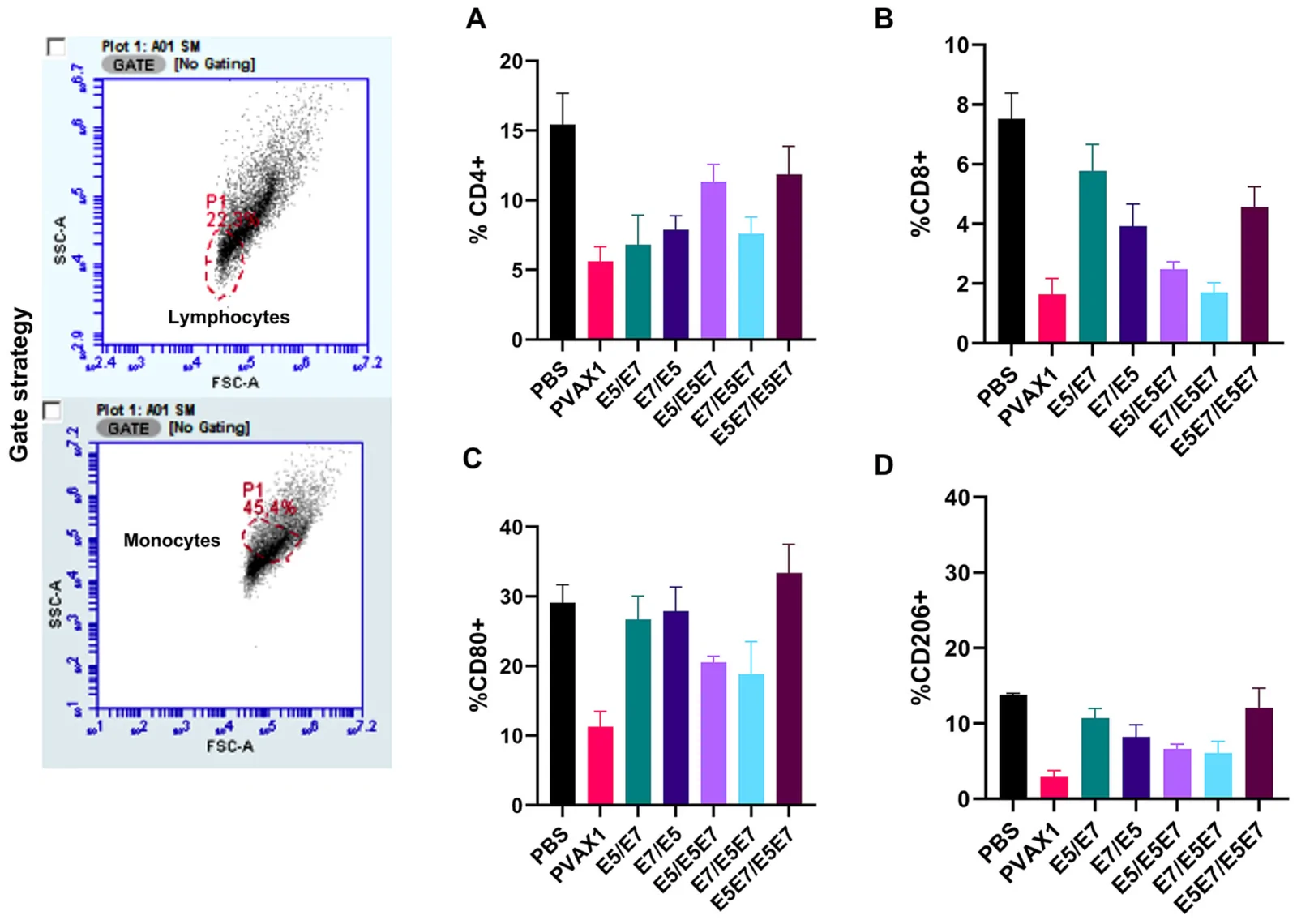
Flow cytometry data showing CD4^+^, CD8^+^, CD80^+^, and CD206^+^ cell subsets across the different experimental groups. In (**A**), the frequencies of CD4^+^, (**B**) CD8^+^, (**C**) CD80^+^, and (**D**) CD206^+^ cell subsets are shown. The left images represent the general gate strategies for the lymphocyte and monocyte populations.

**Figure 9:**
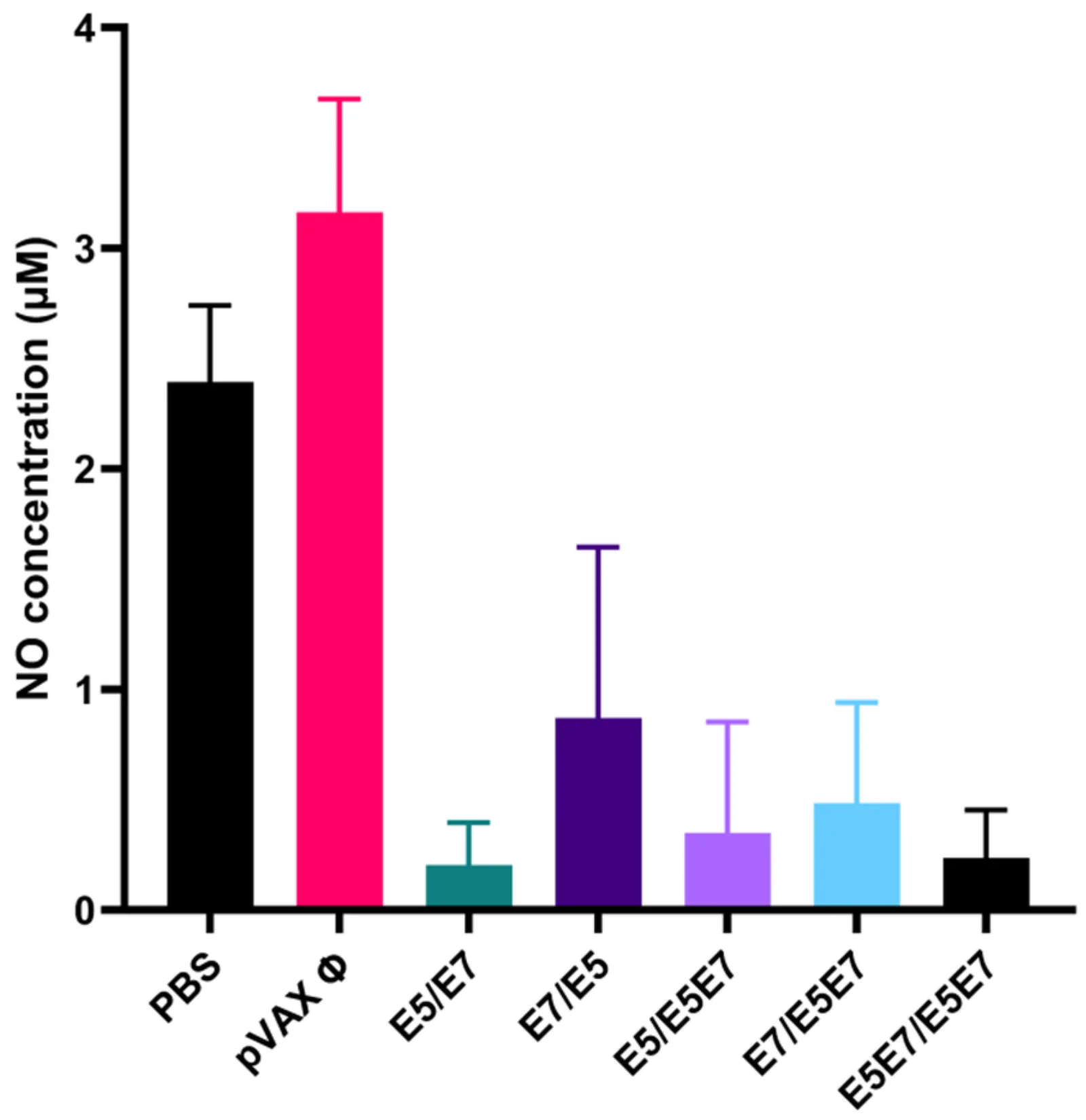
NO concentration measured by the Griess reaction in experimental groups. Bars represent means ± SD. The pVAX group had *n* = 3; all other groups had *n* = 5. Complete pairwise statistical comparisons are provided in Supplementary Table S15.

**Table 1:** Comparison of the predicted physicochemical, toxicity, and allergenicity properties of the HisE5CP and HaE7CP proteins.

Property	HisE5CP	HaE7CP
General Properties		
Number of Amino Acids	324	346
Molecular Weight (kDa)	35.9	37.3
Theoretical pI	8.09	4.72
Stability		
Instability Index (II)	25.55	39.49
Estimated Half-Life (in vitro, Mammalian Reticulocytes)	30 h	30 h
Estimated Half-Life (in vivo, yeast)	>20 h	>20 h
Estimated Half-Life (in vivo, *E. coli*)	>10 h	>10 h
Aliphatic Index	91.26	69.51
Grand Average of Hydropathicity (GRAVY)	0.272	−0.238
Structural Features		
Cysteine Residues	9	10
Predicted toxicity and allergenicity		
ToxinPred 2.0 Prediction	Non-Toxic	Non-Toxic
Allertop 2.0 Prediction	Probably Non-Allergenic	Probably Non-Allergenic

**Table 2:** Thermodynamic parameters of the predicted RNA secondary structures for constructs of interest.

Construct	MFE (kcal/mol)	Ensemble Free Energy (kcal/mol)	Ensemble MFE Frequency	Diversity
HisE5CP	−289.40	−304.52	0.00%	170.30
HaE7CP	−321.70	−337.90	0.00%	209.06
HisE5CP-IRES-HaE7CP	−886.20	−923.70	0.00%	575.40

**Table 3:** Structural validation of HaE7CP and HisE5CP models predicted by AlphaFold 3.

Metric	HaE7CP	HisE5CP
pTM	0.57	0.60
ModFold Global Score	0.4358	0.4705
ModFold *p*-value	1.77 × 10^−3^	8.18 × 10^−4^
Clashscore	1.95	2.37
Ramachandran Favored (%)	92.7	94.0
Ramachandran Allowed (%)	98.0	99.7

**Table 4:** Preservation of predicted MHC-I epitopes following fusion of HPV16 E5 and E7 to CP.

Epitope	Allele	E5wt Percentile Rank	E5CP Percentile Rank	E5wt Processing Score	E5CP Processing Score
IIFVYIPLF	H2-Kb	0.06	0.06	0.06	0.06
LSVSTYTSL	H2-Kb	0.11	0.11	0.13	0.13
STYTSLIIL	H2-Kb	0.13	0.13	0.17	0.17
IVYIIFVYI	H2-Kb	0.17	0.17	0.23	0.23
SAFRCFIVYI	H2-Db	0.63	0.63	0.11	0.11
VCLLIRPLL *	H2-Kb	1.6	1.6	2.2	2.2
YIIFVYIPL *	H2-Kb	0.83	0.83	0.46	0.46
Epitope	Allele	E7wt Percentile Rank	E7CP Percentile Rank	E7wt Processing Score	E7CP Processing Score
RAHYNIVTF *	H2-Db	0.01	0.01	0.03	0.03
RAHYNIVTF	H2-Kb	0.60	0.60	1.8	1.8
YMLDLQPET *	H2-Db	4.1	4.1	6.3	6.3
YMLDLQPET	H2-Kb	8.6	8.6	17	17
STHVDIRTL	H2-Db	0.26	0.26	1.2	1.2
STHVDIRTL	H2-Kb	0.92	0.92	3.4	3.4
TLHEYMLDL	H2-Kb	1.3	1.3	1.7	1.7
DTPTLHEYM	H2-Db	0.46	0.46	1.6	1.6
GTLGIVCPI	H2-Db	2.8	2.8	1.4	1.4
TDLYCYEQL	H2-Db	3.4	3.4	6.7	6.7

* Experimentally validated HPV16 E5 and E7 epitopes are reported in the literature.

**Table 5:** Conservation of representative MHC-II epitopes after fusion of HPV16 antigens to CP.

Antigen	Native Epitope	Fusion Epitope	Core	PR-Native	PR-Fusion	Status
E5	MTNLDTASTTLLACF	NLDTASTTLLACFLL	DTASTTLLA	29	60	Conserved
E5	VLLLWITAASAFRCF	LLWITAASAFRCFIV	ITAASAFRC	30	39	Conserved
E5	VSTYTSLIILVLLLW	LLSVSTYTSLIILVL	YTSLIILVL	88	83	Conserved
E5	ITAASAFRCFIVYII	LLWITAASAFRCFIV	ITAASAFRC	88	39	Conserved
E5	CLLIRPLLLSVSTYT	LIRPLLLSVSTYTSL	LLLSVSTYT	90	81	Conserved
E5	IVYIIFVYIPLFLIH	YIIFVYIPLFLIHTH	FVYIPLFLI	97	81	Conserved
E7	DEIDGPAGQAEPDRA	DEIDGPAGQAEPDRA	IDGPAGQAE	1.9	1.9	Preserved
E7	PAGQAEPDRAHYNIV	PAGQAEPDRAHYNIV	AEPDRAHYN	40	40	Preserved
E7	DLYCYEQLNDSSEEE	DLYCYEQLNDSSEEE	YCYEQLNDS	43	43	Preserved
E7	STHVDIRTLEDLLMG	STHVDIRTLEDLLMG	IRTLEDLLM	48	48	Preserved
E7	PTLHEYMLDLQPETT	PTLHEYMLDLQPETT	YMLDLQPET	67	67	Preserved
E7	MHGDTPTLHEYMLDL	AHGDTPTLHEYMLDL	GDTPTLHEY	93	92	Conserved

## Data Availability

The original contributions presented in this study are included in the article/Supplementary Materials. Further inquiries can be directed to the corresponding author.

## Supplementary Materials

The following supporting information can be downloaded at https://www.mdpi.com/article/10.3390/molecules31193442/s1: Figure S1: Comparative analysis between the previous and post-refinement three-dimensional structures; Figure S2: Humoral responses for constructions with E5 and E7 predicted using C-IMMSIM; Figure S3: Schematic representation of the DNA vaccine constructs used in this study; Figure S4: Western blot analysis of E5CP and E7CP expression in transfected HEK-293T cells. Figure S5: Representative flow cytometry gating strategy for CD4^+^ T lymphocyte identification; Figure S6: Representative flow cytometry gating strategy for CD8^+^ T lymphocyte identification; Figure S7: Representative flow cytometry gating strategy for CD80 expression on myeloid cells; Figure S8. Representative flow cytometry gating strategy for CD206 expression on myeloid cells; Table S1: Primers used for the amplification of cDNA obtained from HEK-293T cells transfected with the vaccine constructs; Table S2: Predicted toxic peptides identified in HisE5CP and HaE7CP and their corresponding MHC-I binding predictions; Table S3: Structural validation of HAE7CP and HisE5CP models predicted by AlphaFold 3; Table S4: MHCI processing table results for E7HPV16 and E7CP; Table S5: MHCI processing table results for E5HPV16 and E5CP; Table S6: MHCI binding table results for E7HPV16 and E7CP; Table S7: MHCI binding table results for E5HPV16 and E5CP; Table S8: Tumor volume at endpoint, tumor-bearing mice, and tumor progression mean; Table S9: Medium weight of the animals during the experiment; Table S10: Histopathological scores; Table S11: Pairwise statistical comparisons for CD80^+^; Table S12: Pairwise statistical comparisons for CD4^+^; Table S13: Pairwise statistical comparisons for CD8^+^; Table S14: Pairwise statistical comparisons for CD206^+^; Table S15. Pairwise statistical comparisons for nitric oxide production. Table S16. Pearson correlation between nitric oxide (NO) levels and tumor volume in the different experimental groups (*n* = 33).

## Abbreviations

The following abbreviations are used in this manuscript:

APC	Antigen-Presenting Cell
CEUA	Comissão de Ética no Uso de Animais (Animal Ethics Committee)
CONCEA	Brazilian National Council for the Control of Animal Experimentation
CP	Coat Protein (of Potato Virus X)
DNA	Deoxyribonucleic Acid
FBS	Fetal Bovine Serum
FITC	Fluorescein Isothiocyanate
H&E	Hematoxylin and Eosin
HEK-293T	Human Embryonic Kidney 293T cells
HLA	Human Leukocyte Antigen
HPV	Human Papillomavirus
HPV16	Human Papillomavirus Type 16
IEDB	Immune Epitope Database
IL	Interleukin
IRES	Internal Ribosome Entry Site
MFE	Minimum Free Energy
MHC	Major Histocompatibility Complex
NO	Nitric Oxide
PBS	Phosphate-Buffered Saline
PCR	Polymerase Chain Reaction
PE	Phycoerythrin
pI	Isoelectric Point
pTM	Predicted Template Modeling Score
PVX	Potato Virus X
RNA	Ribonucleic Acid
RT-PCR	Reverse Transcription Polymerase Chain Reaction
SPF	Specific pathogen-free
