# Supplementary material for: Heterologous Antigen Regimen and Bicistronic DNA Vaccination Targeting HPV16 E5 and E7 Oncoproteins: Immunoinformatics and Preclinical Antitumor Efficacy
Source: Molecules. 2026 Sep 27;31(19):3442. doi: 10.3390/molecules31193442 (PMC13666130; doi:10.3390/molecules31193442)
Supplement: Supplementary file 1 [file molecules-31-03442-s001.zip › Supplementary Materials Figures.pdf]

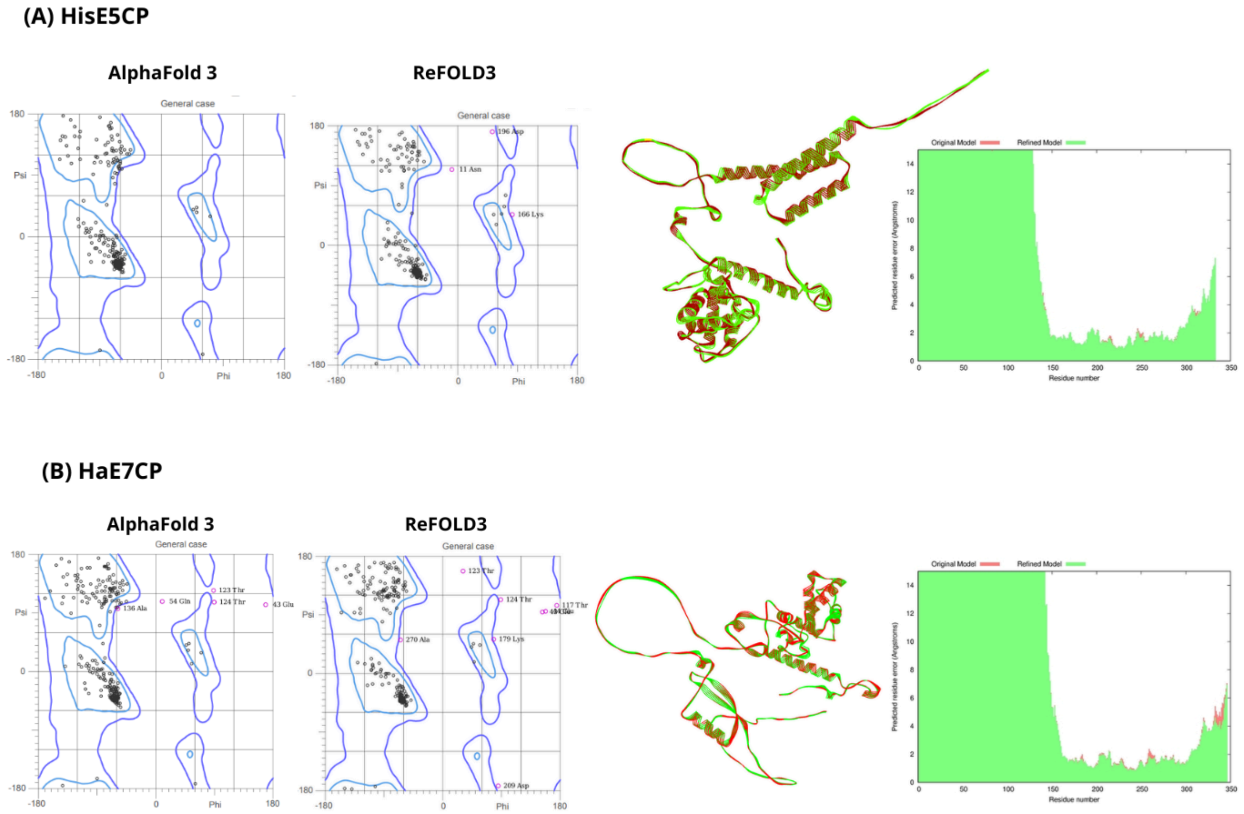


**Figure S1**. Comparative analysis between the previous and post-refinement three-dimensional structures. (A) Shows the Ramachandran plot for HisE5CP from AlphaFold 3 and after refinement with ReFOLD3. (B) Shows the Ramachandran plot for HaE7CP from AlphaFold 3 and after refinement with ReFOLD3.

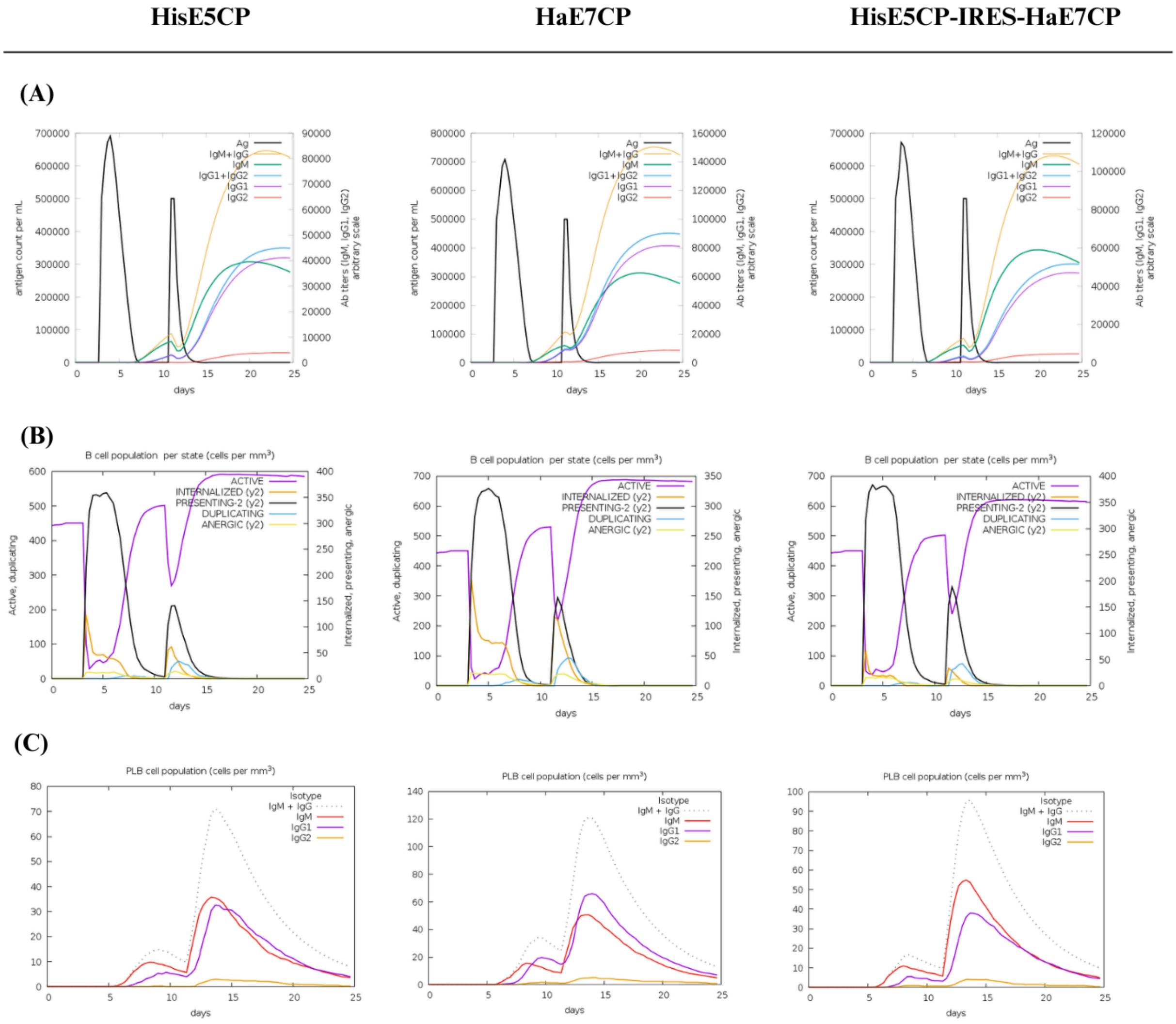


**Figure S2**. Humoral responses for constructions with E5 and E7 predicted using C-IMMSIM within a human HLA framework. In (**A**) shows the antibodies and immunoglobulins. In (**B**) B lymphocytes population per entity-state showing counts for active, presenting on class-II, internalized the Ag, duplicating and anergic. (**C**) shows plasma B lymphocytes count sub-divided per isotype (IgM, IgG1 and IgG2).

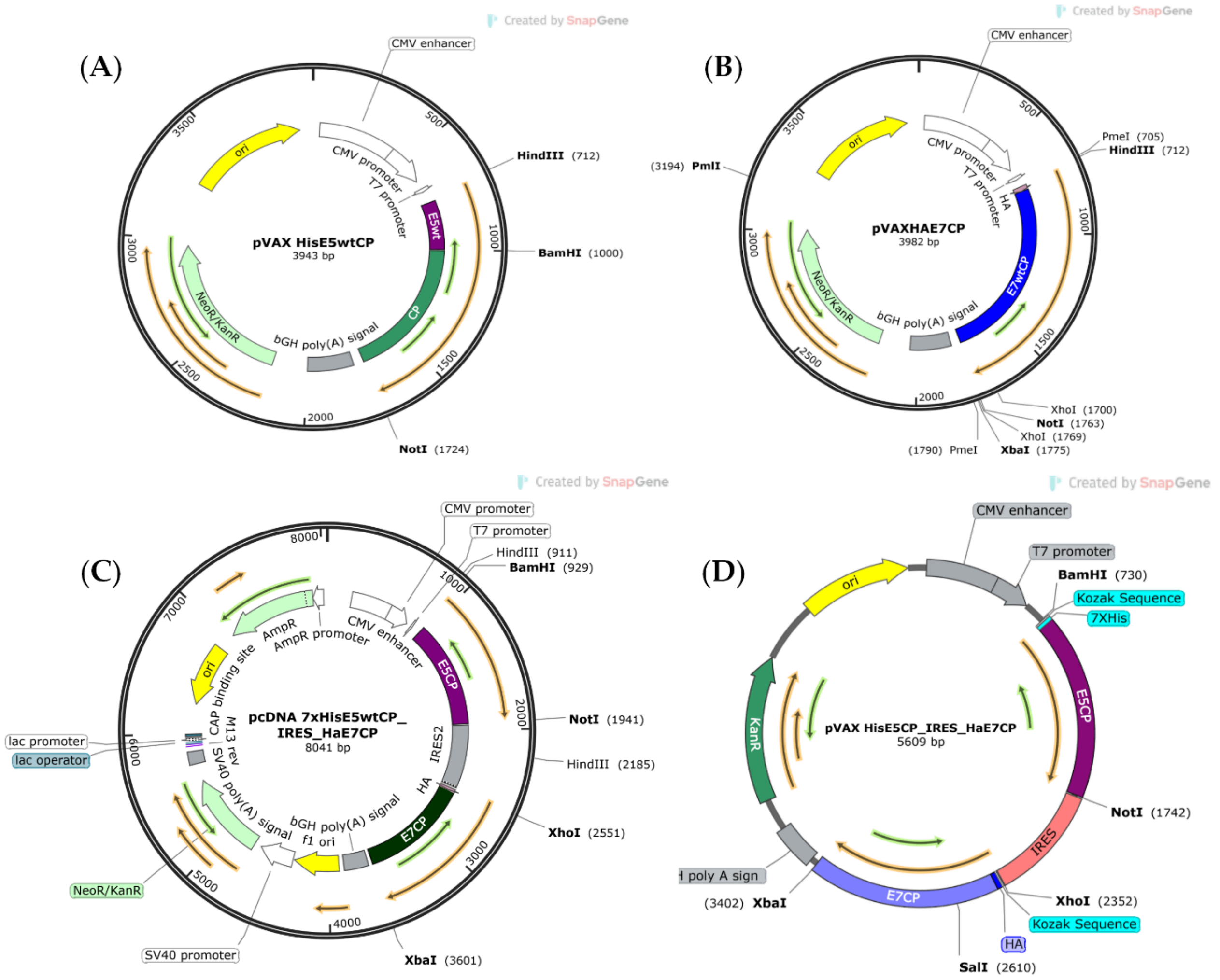


**Figure S3**. Schematic representation of the DNA vaccine constructs used in this study. (**A**) pVAX-E5CP: monocistronic construct encoding the E5 gene fused to CP (E5CP). (**B**) pVAX-E7CP: monocistronic construct encoding the E7 gene fused to CP. (**C**) Synthesized plasmid pcDNA3.1-E5CP-IRES-E7CP. (**D**) pVAX-E5CP-IRES-E7CP (E5E7): bicistronic cassette subcloned into the pVAX.1 base vector, used for in vivo DNA vaccination.

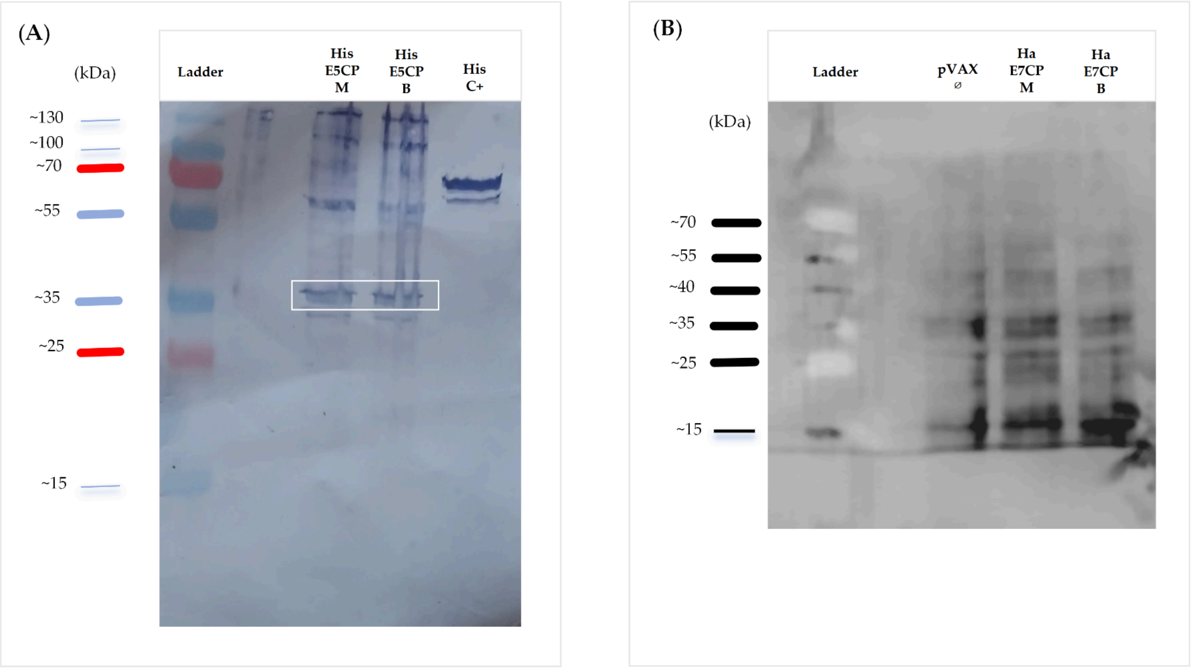


**Figure S4.** Western blot analysis of E5CP and E7CP expression in transfected HEK-293T cells. (A) Detection of His-tagged E5CP using an anti-polyhistidine antibody, visualized by colorimetric detection with NBT/BCIP. (B) HA-tagged E7CP was detected using an anti-HA antibody and visualized by chemiluminescence with ECL substrate. In both panels, M indicates monocistronic constructs, and B indicates the bicistronic construct. E5CP migrated anomalously and was also detected as higher-molecular-weight multimers, consistent with its cysteine-rich and hydrophobic nature. For E7CP, bands could not be reliably assigned because the empty vector control showed the same background signal. Therefore, these results should be interpreted as inconclusive for protein-level expression of the chimeric constructs.

# CD4+ Lymphocyte Gates

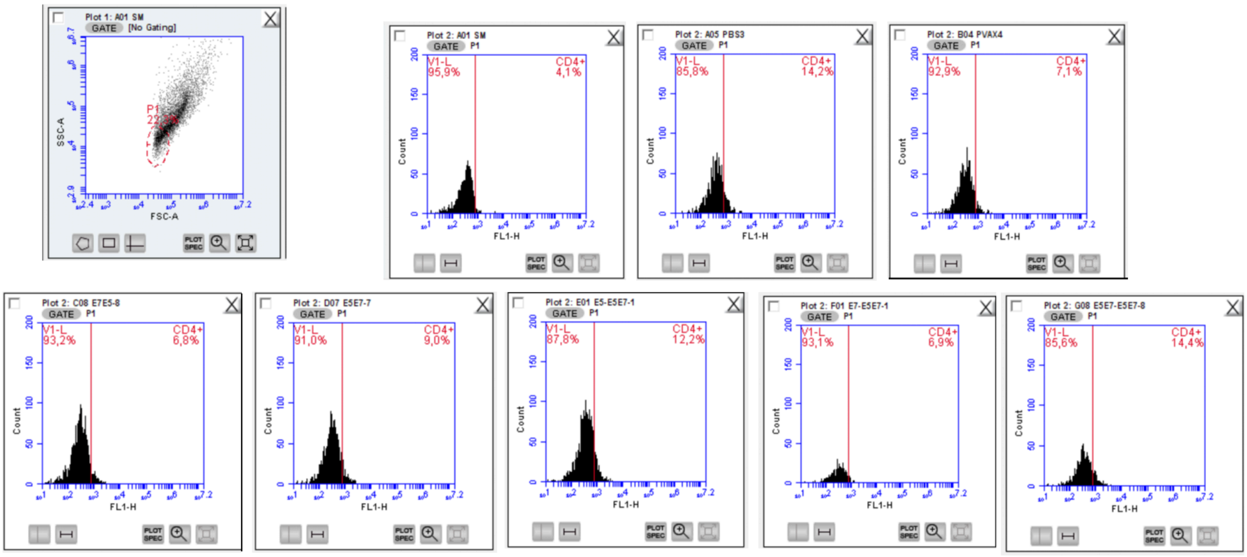


**Figure S5**. Representative flow cytometry gating strategy for CD4+ T lymphocyte identification.

# CD8+ Lymphocyte Gates

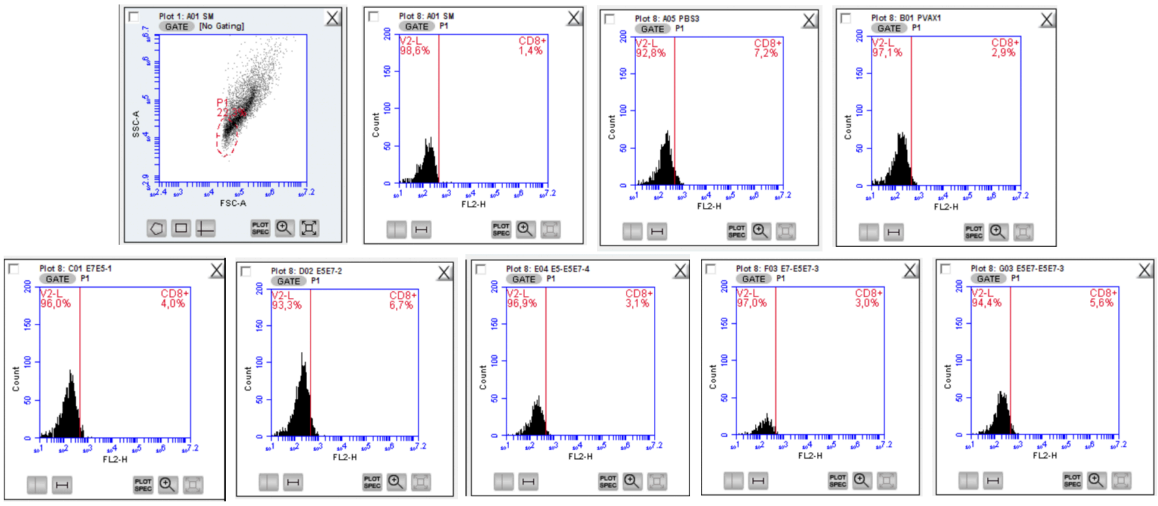


**Figure S6**. Representative flow cytometry gating strategy for CD8+ T lymphocyte identification.

## CD80+ Lymphocyte Gates

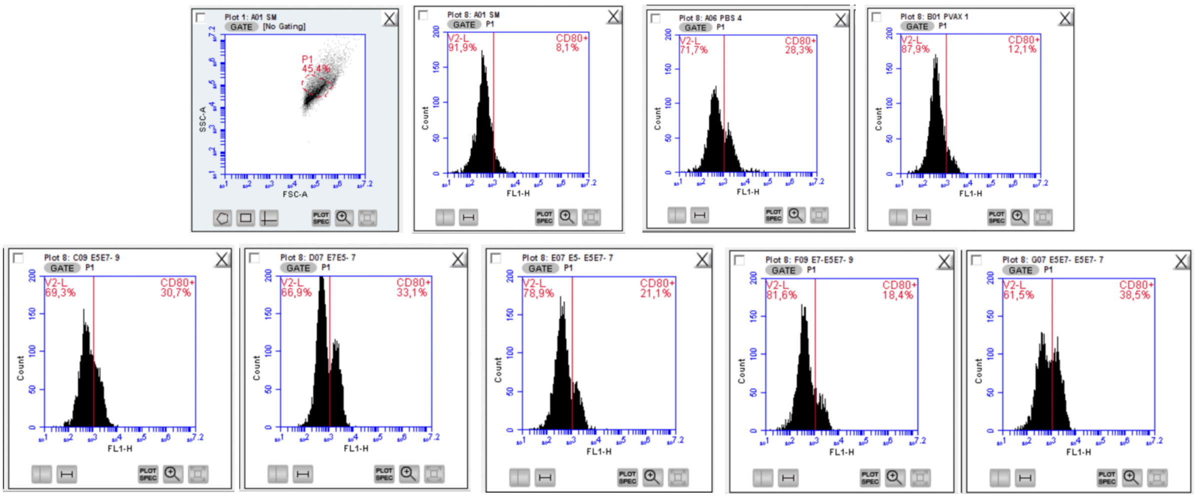

**Figure S7**. Representative flow cytometry gating strategy for CD80+ expression on myeloid cells.

## CD206+ Lymphocyte Gates

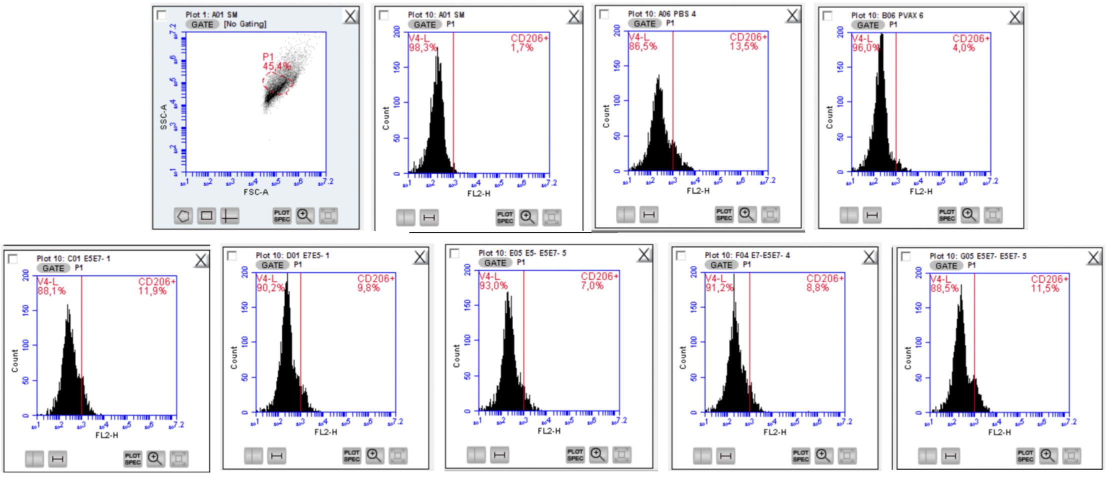

**Figure S8**. Representative flow cytometry gating strategy for CD206+ expression on myeloid cells.
